# In silico modeling of the effects of alpha-synuclein oligomerization on dopaminergic neuronal homeostasis

**DOI:** 10.1186/1752-0509-8-54

**Published:** 2014-05-13

**Authors:** Eleftherios Ouzounoglou, Dimitrios Kalamatianos, Evangelia Emmanouilidou, Maria Xilouri, Leonidas Stefanis, Kostas Vekrellis, Elias S Manolakos

**Affiliations:** 1 Department of Informatics and Telecommunications, Graduate Program “Information Technologies in Medicine and Biology”, National and Kapodistrian University of Athens, Athens 15784, Greece; 2 In Silico Oncology Group, Laboratory of Microwaves and Fiber Optics, Institute of Communication and Computer Systems, School of Electrical and Computer Engineering, National Technical University of Athens, 9 Iroon Polytechniou, Zografou, 15780 Athens, Greece; 3 Division of Developmental Biology, Biomedical Research Foundation of the Academy of Athens, 4 Soranou Efesiou, Athens 11527, Greece; 4 Division of Basic Neurosciences, Biomedical Research Foundation of the Academy of Athens, 4 Soranou Efesiou, Athens 11527, Greece; 5 Second Department of Neurology, University of Athens Medical School, Athens, Greece

**Keywords:** Parkinson’s disease, Alpha-synuclein, Neurodegeneration, Biomolecular reaction networks modeling, In silico experiments

## Abstract

**Background:**

Alpha-synuclein (ASYN) is central in Parkinson’s disease (PD) pathogenesis. Converging pieces of evidence suggest that the levels of ASYN expression play a critical role in both familial and sporadic Parkinson’s disease. ASYN fibrils are the main component of inclusions called Lewy Bodies (LBs) which are found mainly in the surviving neurons of the substantia nigra. Despite the accumulated knowledge regarding the involvement of ASYN in molecular mechanisms underlying the development of PD, there is much information missing which prevents understanding the causes of the disease and how to stop its progression.

**Results:**

Using a Systems Biology approach, we develop a biomolecular reactions model that describes the intracellular ASYN dynamics in relation to overexpression, post-translational modification, oligomerization and degradation of the protein. Especially for the proteolysis of ASYN, the model takes into account the biological knowledge regarding the contribution of Chaperone Mediated Autophagy (CMA), macro-autophagic and proteasome pathways in the protein’s degradation. Importantly, inhibitory phenomena, caused by ASYN, concerning CMA (more specifically the lysosomal-associated membrane protein 2a, abbreviated as Lamp2a receptor, which is the rate limiting step of CMA) and the proteasome are carefully modeled. The model is validated by simulation studies of known experimental overexpression data from SH-SY5Y cells and the unknown model parameters are estimated either computationally or by experimental fitting. The calibrated model is then tested under three hypothetical intervention scenarios and in all cases predicts increased cell viability that agrees with experimental evidence. The biomodel has been annotated and is made available in SBML format.

**Conclusions:**

The mathematical model presented here successfully simulates the dynamic phenomena of ASYN overexpression and oligomerization and predicts the biological system’s behavior in a number of scenarios not used for model calibration. It allows, for the first time, to qualitatively estimate the protein levels that are capable of deregulating proteolytic homeostasis. In addition, it can help form new hypotheses for intervention that could be tested experimentally.

## Background

Parkinson’s disease (PD) is a degenerative disorder of the central nervous system caused by the selective loss of dopaminergic neurons (DN) in the substantia nigra (SN), a region of the midbrain. The neuronal loss, which can be as high as approximately 70%, is accompanied by the loss of the physiologic functionality of the dopaminergic system, leading to the emergence of severe motor symptoms, such as bradykinesia, muscular rigidity and resting tremor. A central pathological hallmark of PD is the formation of inclusions, known as Lewy bodies (LBs), caused by the accumulation of aggregated proteins in various regions of the brain and importantly in the surviving neurons of the substantia nigra. The main components of LBs are aggregates of the protein alpha-synuclein (ASYN), a 140 amino acid pre-synaptic protein that has been genetically linked to Parkinson’s disease. In particular, missense mutations (that lead to a different amino acid sequence) in the ASYN gene (SNCA) have been correlated with familial PD [1-3]. However, the multiplication of SNCA has been also linked to the disease and polymorphisms that cause modification of the ASYN transcription have been shown to increase the risk for sporadic PD development, suggesting that even the Wild Type (WT) form of ASYN, if overexpressed, could contribute to the development of PD [4-7].

Numerous experimental findings have been published in recent years concerning ASYN-related mechanisms that may be responsible for the observed neurodegeneration. For example, a correlation between the over-expression of WT ASYN and the observed cell loss has been shown in SH-SY5Y cells [8], a human neuroblastoma cell line that upon treatment with retinoic acid assumes a neuronal phenotype; SH-SY5Y cells have often been used to model PD following various toxicological or molecular insults. Moreover, a main characteristic of ASYN is its propensity to form soluble oligomers, which are thought to be the intermediate steps in the formation of the fibrils found in LBs [9]. This oligomerization capacity has been observed both in vitro [10] and in vivo [8,11]. There is evidence that the soluble oligomers are the toxic species and not the insoluble fully fibrillar forms of ASYN [9]. Moreover, it is believed that the formation of LBs could be a protective mechanism triggered by neurons in order to isolate the toxic soluble oligomers from the other cytoplasmic species and organelles [9,12]. This is also in agreement with the detection of LBs in the remaining surviving neurons in the SN. Supporting these hypotheses, it has been shown that oligomers, in the absence of fibrils or aggregates, are capable of disrupting the homeostasis of differentiated WT ASYN-overexpressing SH-SY5Y cells, leading to their degeneration. This was shown through the use of the oligomer-stabilizing agent Scyllo-inositol, which reversed the cytotoxic phenomena caused by the over-expression of WT ASYN [8]. This suggests a correlation between ASYN oligomers and cell death. Furthermore, since the main pathological phenomena of PD occur in the dopaminergic neurons of the SN, a relationship between the presence of dopamine (DA) and the development of the disease could hypothetically exist. Numerous in vitro or in vivo studies have provided evidence (for a comprehensive review see [13]), that DA can modulate the oligomerization process of ASYN. More specifically, it is shown that the potential modification of ASYN by DA, inhibits (or at least delays) the formation of ASYN fibrils and the aggregation of the protein, leading to the accumulation of soluble oligomers. Furthermore, this modification was found to have a causal role in the observed inhibition of WT ASYN on Chaperone Mediated Autophagy (CMA) [14,15], one of the major pathways contributing to the proteolysis of ASYN [14,16,17]. A central molecular entity in the CMA proteolytic pathway is the lysosomal-associated membrane protein 2a (Lamp2a) receptor. CMA substrates are transferred to Lamp2a, with the help of cytosolic chaperones, and then are internalized into the lysosomes in order to be degraded. One of the known substrates of CMA is ASYN. However, as shown in [15], the DA-modified forms of ASYN, despite binding to the Lamp2a receptor, do not get internalized into the lysosomes, and thus cause an aberrant occupation of the receptor which inhibits the normal function of CMA. This inhibition of CMA functionality was shown to be correlated with the observed cell death of differentiated SH-SY5Y cells [14]. The involvement of DA in WT ASYN-mediated cytotoxicity in this model was confirmed, since the repression of DA production reversed the dysregulation of lysosomal proteolysis and, more importantly, cell loss [14]. Altogether, the above data suggest that the DA-mediated modification of ASYN may have a central role in the induction of neurodegeneration. As for the contribution of other proteolytic machineries in the degradation of ASYN, there is significant evidence that both macroautophagy [16] and the proteasome [18-20] contribute to the removal of various ASYN species (from monomers to oligomers). However, it should be mentioned that in our experimental setting (SH-SY5Y cell line) the contribution of the proteasome to the degradation of monomeric ASYN was not found to be significant [16]. However, it is also believed that the latter mechanism could be impaired by specific ASYN species of higher molecular weight (oligomeric and fibrillar) [18,20-24].

It is worth mentioning that many other ASYN-related pathogenic phenomena have been reported in the literature. Amongst others, ASYN overexpression is found to modulate neuronal calcium homeostasis [25] and its oligomers could lead to an increase of calcium influx [26]. Also, these ASYN species are believed to be able to disrupt various types of cellular membranes, such as those of mitochondria [8,27] or even the plasma membrane [28,29].

**Figure 1:**
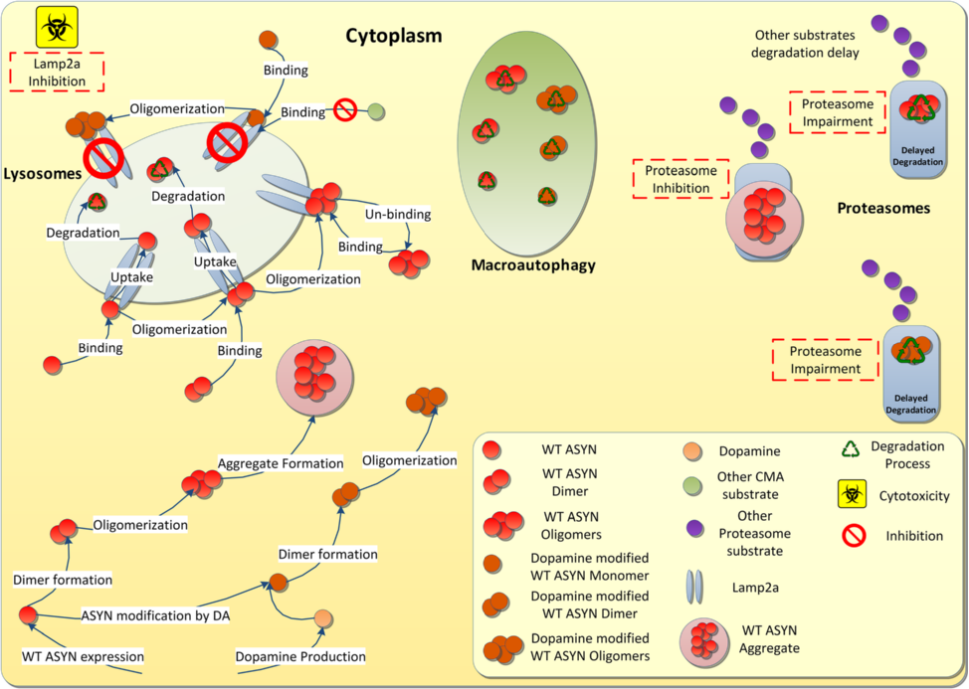
**Modeled ASYN dynamics in the cytoplasm.** Following protein expression and oligomerization, monomers and dimers bind to Lamp2a and either continue to oligomerize or release the receptor by entering the lysosome and undergoing degradation. Oligomers (i.e. Low Molecular Weight (LMW) species up to nonamers in this study) also bind to Lamp2a but they do not enter the lysosome and they do not affect the degradation of other substrates. On the other hand, dopamine-modified monomers bind to Lamp2a, oligomerize without entering the lysosome and inhibit the functionality of the receptor. Species up to oligomers of WT and modified ASYN are degraded via macroautophagy. Oligomers of ASYN are targeted for degradation by the proteasome, but also can impair its function. Aggregates of ASYN (i.e. High Molecular Weight (HMW) species beyond nonamers in this study) can inhibit the proteasome’s function (not observed in our experimental setting, see text for details).

Despite the accumulated knowledge concerning the involvement of ASYN in molecular mechanisms underlying the development of PD, there is substantial information missing before a cure can be found. Systems Biology approaches could play a crucial role in integrating and further utilizing the current knowledge and the first PD-related models have already appeared. In [20] a mathematical model of the effects of ASYN oligomerization on cells’ homeostasis has been proposed, however, this model focused only on Proteasome dynamics. In [24] an existing model of protein aggregation regulation via UCH-L1 has been extended to simulate this process with ASYN. Although several of the phenomena described above, such as CMA and proteasome inhibition, have been modeled in [24], the role of DA has not been taken into account and efforts have focused primarily on the formation of inclusions, whose role in mediating neurodegeneration is debatable. In a more recent study a dynamic model that linked mutated ASYN, mitochondrial function, and glutathione (GSH) metabolism has been proposed [12]. However, the focus of this study was on ASYN aggregation phenomena in the presence or absence of oxidative stress and not on DA-modified ASYN dynamics. The scope was the exploration of possible points of drug intervention for reducing cell apoptosis following ASYN aggregation.In the present study, we sought to create a holistic model capable of simulating the dynamics of ASYN overexpression and oligomerization, and integrating the fundamental lysosomal and proteasomal degradation pathways, as well as the modifications conferred by DA. In this model, we have correlated these phenomena to the aberrant function of intracellular ASYN. To develop the model, we used recent experimental findings on the role of ASYN in PD development, combined with the experience gained by previous ASYN-related modeling efforts. In our modeling approach, we focused on the dynamics of DA-modified ASYN species and on phenomena previously correlated with neurotoxicity (such as CMA inhibition via ASYN-mediated Lamp2a over-occupation). Using data derived by experimentation, mainly from the SH-SY5Y cell line, we managed to reproduce the experimental system’s behavior in silico. This allowed us to investigate, by stochastic simulation, the neuroprotective potential for a number of hypothetical interventions in the system. The phenomena and the interactions that were modeled are pictorially summarized in Figure 1 and are discussed in detail in the Model development section of Results and discussion.

## Results and discussion

### Modeling approach and objectives

To describe the intracellular ASYN dynamics we developed a biomolecular reactions network capturing the interactions of modeled species. This is then translated to a set of ordinary differential equations (ODEs). Next, we performed experimental measurements of ASYN species levels (monomer, dimer, oligomers) after inducibly over-expressing ASYN by a tet-off system (see Methods and [8] for details) and the collected data was used to fit model parameters. By calibrating the model we aimed to reproduce the dynamics of over-expressed WT ASYN, its oligomerization and its interplay with CMA (focusing on Lamp2a-related phenomena), proteasome and macroautophagy pathways. Using computer simulations, we sought to investigate, in silico, the roles of these pathways as well as the contribution of DA in the aberrant function of ASYN. The calibrated model was finally used to predict the biological system’s behavior in a number of hypothetical intervention scenarios, none of which was previously used for model calibration.

### Model development

The developed model comprises of five major components (modules) which interact with each other: ASYN production and modification oligomerization, degradation by CMA, degradation by macroautophagy and degradation by the proteasome.

**Figure 2:**
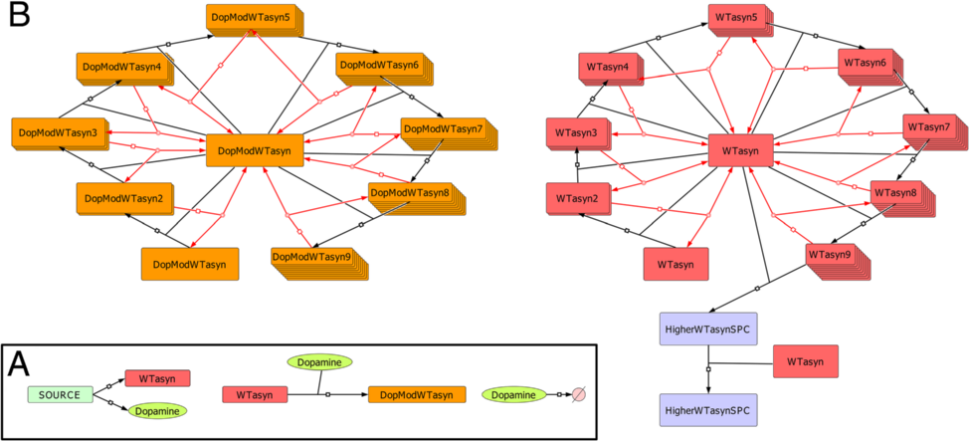
**Graphical representation of the model structure in SBGN for: (A) ASYN and DA production and (B) ASYN oligomerization and modification sub-models. (A)** WT ASYN (WTasyn) and DA (Dopamine) are produced by SOURCE (see text for details). The WT ASYN is modified by DA to form the modified ASYN Monomer (DopModWTasyn). The DA species are degraded by a simple degradation reaction. **(B)** WT (WTasyn-WTasyn9) and DA-modified (DopModWTasyn-DopModWTasyn9) can form oligomers of higher order (black colored reactions) by the addition of a monomer. Moreover, oligomers could dissociate to oligomers of a lower order by freeing a monomer species (red colored reactions). Only WT ASYN species can form HMW species (HigherWTasynSPC).

**Figure 3:**
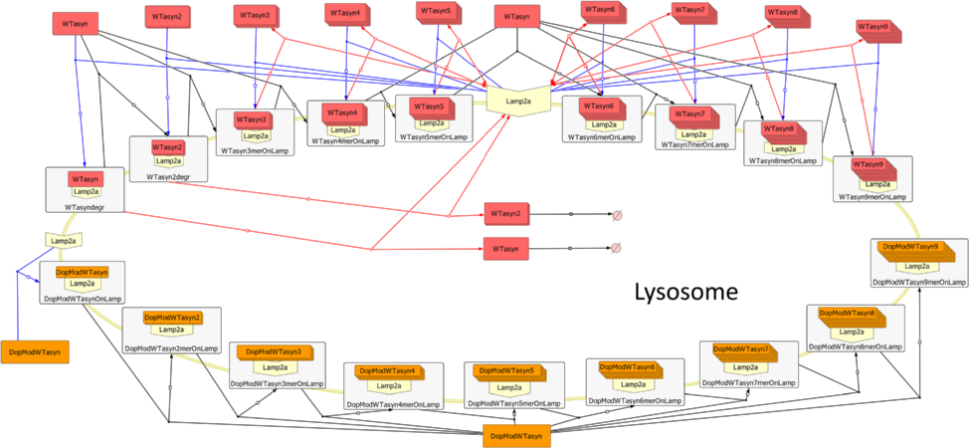
**Graphical representation of the model structure in SBGN for the Lysosome-CMA compartment.** Upper part of the figure: The monomer and dimer WT ASYN species (WTasyn & WTasyn2) bind to the Lamp2a receptor (blue colored reactions) and are then inserted to the lysosome in order to be degraded. Oligomers of WT ASYN (WTasyn3-WTasyn9) can also bind to Lamp2a (blue colored reactions) and although are not inserted into the lysosome, this binding is reversible (red colored reactions). Finally, the WT ASYN can also oligomerize on top of Lamp2a (black colored reactions). Lower part of the figure: DA-modified ASYN monomeric species (DopModWTasyn) bind to Lamp2a receptor (blue colored reaction) in an irreversible manner. Moreover, the modified species can also form oligomers on top of Lamp2a (black colored reactions), further suppressing this repressor.

**Figure 4:**
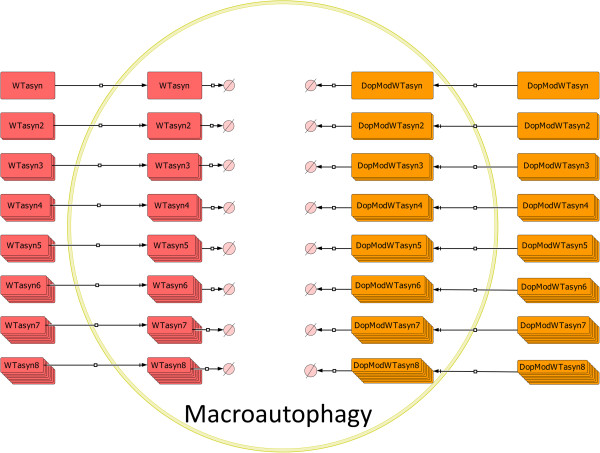
**Graphical representation of the model structure in SBGN for the Macroautophagy compartment.** Both WT (WTasyn) and DA-Modified (DopModWTasyn) ASYN species up to 8mers are degraded by Macroautophagy. After they are isolated from the cytosol, they are degraded by simple degradation reactions.

**Figure 5:**
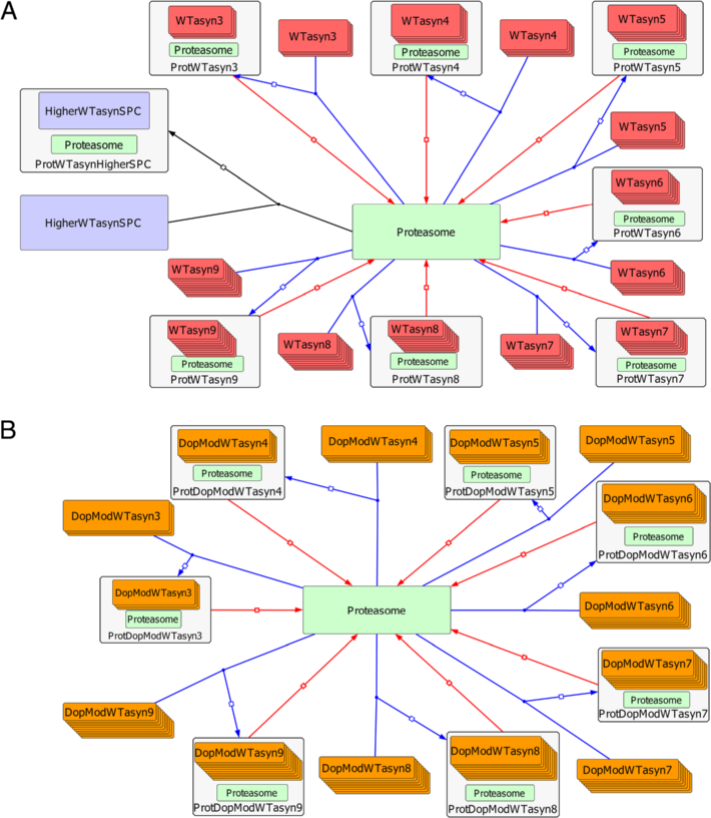
**Graphical representation of the model structure in SBGN for the Proteasome related ASYN dynamics sub-model.** Both WT (WTasyn, Figure 5A) and DA-modified (DopModWTasyn, Figure 5B) ASYN species bind to the Proteasome (blue colored reactions) and are then degraded by releasing it (red colored reactions). Also HMW ASYN species can bind to the Proteasome, however in an irreversible way.

In a similar approach to [24], it is assumed that both WT ASYN and DA are produced at a constant rate from a “source”. This entity (which has constant/unchanged levels) accounts for the protein synthesis machinery of the cell. We also assumed that the production rates of ASYN and DA are a combination of their transcription and translation rates. This assumption was made due to restrictions imposed by the experimental protocol used to produce the data. More specifically, in our experimental system, WT ASYN was expressed by a tet-off system (ectopic expression) [8], making the modeling of the exact procedure extremely complicated since both endogenous and ectopic expression should be taken into account. Thus, a complex combinatorial process was modeled by introducing the “source” entity and related rates. Similarly, and to overcome the complex DA expression dynamics in neuronal cells, DA was assumed to be produced by the same source. Figures 2, 3, 4, 5 provide a graphical depiction of all biomolecular interactions in the system. The distinct sub-models given in these figures when taken all together constitute the entire model and are defined inside a shared compartment, which refers to the cell’s cytosol (see Methods). The consolidated graph is given in Additional file 1. It was produced using the Systems Biology Graphical Notation (SBGN) standard [30] and it was implemented in CellDesigner [31,32]. Moreover, a pure SBML version of the model, derived from the Additional file 1 in CellDesigner, is given in Additional file 2.

As shown in Figure 2A, the released DA reacts with WT ASYN to produce a new modified molecular entity. At the same time, part of DA is turned over at a constant rate. The oligomerization of ASYN and its DA-modified form is modeled as follows (Figure 2B): the formation of oligomeric species (black colored reactions) and their dissociation (red colored reactions) are executed by adding or removing a monomer, respectively. Although in vitro detailed modeling studies of ASYN aggregation, such as the one performed in [33], do exist, the aforementioned DA-mediated modification of the procedure has not previously taken into account. Thus, the simplest possible way to model the oligomerization procedure (addition of a monomer in every step) was adopted so as to avoid further unverified assumptions.

Moreover, Figure 2B suggests that the oligomerization of WT ASYN and its modified form take place in parallel. They follow the same motif but only non-modified WT ASYN can form higher molecular weight species (HMW: higher than nonamers). These forms may be considered as aggregates and they can increase in molecular weight with the addition of monomer units, but lack the ability to decompose themselves (adopting the assumption also made in [24]). Although in [8,18] there were no HMW species detected in SH-SY5Y cells (only soluble oligomers having molecular weights ranging from dimers to nonamers were detected), it was nevertheless deemed appropriate to include them in our model. This would allow us to capture their behavior as described in other biological and modeling studies [20,21,23,24] and retain the ability to test different hypotheses regarding their role in neurodegeneration. However, it should be mentioned that during the parameter estimation procedure (see Methods), specific constraints have been set to ensure that HMW species levels remain insignificant to meet our experimental findings.

In [24] all species higher than hexamers were considered insoluble, while in our model the same applies only to HMW species higher than nonamers. Our approach is based on the experimental findings in [8] that show soluble forms of ASYN with molecular weight of approximately 130 kDa. Moreover, it has been suggested [34] that the involvement of DA in the oligomerization process of ASYN mainly affects species with molecular weights between ~15 kDa and ~150 kDa, matching the molecular weight spectrum of ASYN monomers to ASYN nonamers. Based on these findings we have made the assumption that DA-modified ASYN can form complexes up to nonamers and species of higher order should only be a product of the unmodified ASYN oligomerization process.

In the next system component, the lysosome (Figure 3), we captured the known interactions related to the degradation of ASYN by CMA. Based on [15], monomers and dimers of un-modified ASYN bind to the Lamp2a receptor and form a complex (upper part of the figure, blue colored reactions). As soon as they enter the lysosome they are degraded into amino acids without affecting the normal functionality of CMA degradation. ASYN oligomers (trimers up to nonamers) also bind to Lamp2a (upper part of the figure, blue colored reactions) but they are quickly released before entering the lysosome (upper part of the figure, red colored reactions). During the binding of oligomers to Lamp2a, the receptor is not allowed to bind to any other entities (other Lamp2a substrates). However, as shown in [15], this repression is not permanent. We have also modeled the ability of ASYN which is bound to Lamp2a to form low molecular weight (LMW) oligomers, up to nonamers [15] (upper part of the figure, black colored reactions).

There is evidence [15] that monomers of DA-modified ASYN show much higher affinity to Lamp2a in comparison with species of un-modified ASYN. Thus, for reasons of simplicity, we have considered in our approach that only monomers of the DA-modified protein can directly form complexes with the receptor. Any oligomers bound to the receptor are formed on site as described above (lower part of Figure 3, black colored reactions). In contrast to the unmodified monomer and dimer species, the complex does not enter the lysosome, meaning that the binding to Lamp2a is irreversible and it results in the suppression of the receptor. Oligomers formed by DA-modified ASYN species bound on Lamp2a also lead to permanent suppression of the receptor.

ASYN species can also be degraded via macroautophagy, a distinct type of the autophagic pathway in which cytoplasmic components are degraded by lysosomes. In this pathway, cytosolic regions are sequestered into autophagosomes that deliver their contents to late endosomal and lysosomal compartments for degradation. These species cannot interact with other entities in the surroundings, e.g., they cannot form oligomers by reacting with cytosolic ASYN species. In this sense, a new compartment was incorporated into the system to account for the autophagosomes formed during the action of the macroautophagic proteolytic machinery. As shown in Figure 4, the procedure has been significantly simplified and the complex dynamics of autophagosome formation and its transition to autophagolysosome have been omitted. Here, the autophagosome is responsible for the internalization and degradation of ASYN and its modified by DA forms via macroautophagy. The modeling procedure is the same for both forms of the protein and for all species from monomers to octamers [18].

The final component of the model, shown in Figure 5, incorporates the available knowledge regarding the interaction of ASYN with the proteasome. Here, again, two parallel processes were modeled; one for the WT protein (Figure 5A) and one for its DA modified form (Figure 5B). Experimental data suggest that this proteolytic machinery has specificity to the ASYN species that it can degrade. Thus, only trimers to nonamers of both forms are targeted to the proteasome (blue colored reactions) which is released after they get degraded [18] (red colored reactions). However, due to the high numbers and structural complexity of this type of species, it is believed that the proteasome gets over-occupied for long periods of time, leading to a delay on the degradation of other proteasome substrates, a process that could be considered as proteasome impairment [18]. On the other hand, higher molecular weight species of WT ASYN could bind to the proteasome irreversibly (black colored reaction), thus suppressing its function indefinitely. However, it should be mentioned that since higher molecular weight ASYN species were not detectable in our experimental system (neither in the cytoplasm nor bound to proteasomes [18]), during the model parameterization procedure we tried to keep the levels of these species within ranges that could be considered experimentally undetectable (see Methods).

### Mathematical definition of the model, initial parameter values and model calibration using experimental data

**Figure 6:**
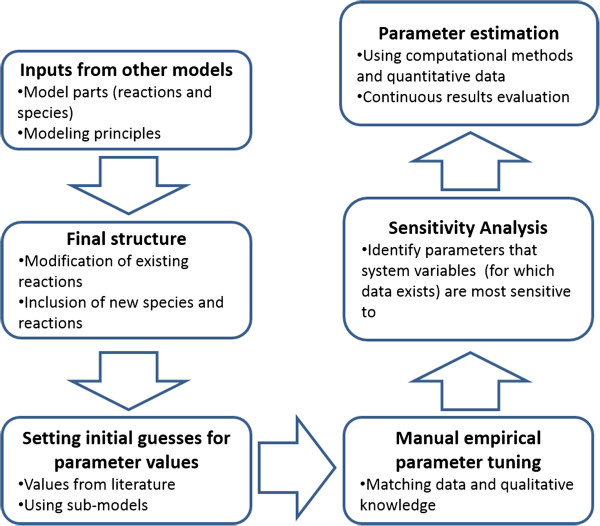
Model development and calibration procedure.

In order to build a model that could be simulated (executable model), we first needed to define the kinetic rate laws governing the model reactions. Moreover, in order for the model to converge to the observed experimental behavior of the system, the underlying parameter values of the kinetic laws had to be precisely predicted. Prior to that, estimated starting points of the parameter search space for these values had to be defined. The latter step consisted of an extensive search in the relevant literature while the former step was implemented using stochastic global optimization algorithms. A detailed description of all steps is provided in the Methods. Moreover, a depiction of the model’s mathematical definition and calibration steps is provided in Figure 6.

### Model validation

**Figure 7:**
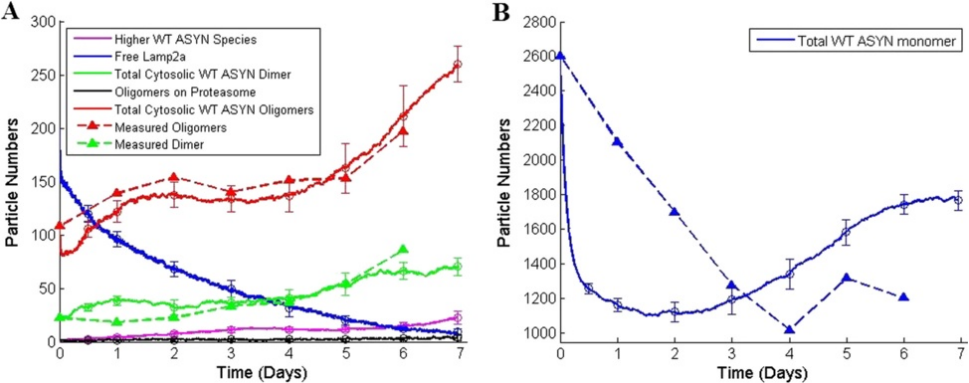
**Average output curves of ten stochastic simulations of the model for the WT over-expression system. (A)** Total amount of ASYN oligomeric (red) and dimeric species (green) are very similar when compared to quantitative experimental data (dashed lines). Levels of Lamp2a, HMW species, oligomers in proteasome also match the available qualitative knowledge. **(B)** Although simulation output for WT ASYN monomers did not accurately reproduce the experimental data, it did give a good indication of the observed phenomena regarding the trends: initial drop followed by a weak recovery.

The next step of the in silico modeling process was to validate the model by testing if it can reproduce the behavior of the biological system and its components as observed in the laboratory. For this purpose, we used the Gibson & Brook stochastic simulation algorithm (SSA) [35] which is faster when compared to other versions of exact SSA algorithms [36]. Figure 7, shows simulation of the model over a 7 day (600,000 sec) “lab time” course and the average species trajectories over 10 simulation runs along with the standard deviation bars at the time points where experimental data were collected. Experimental data and their trends are also plotted (dashed lines) in the same figure. Details pertaining to the nature of the experimental data and its collection are provided in the Methods section.

As shown in Figure 7A, simulated levels of the total amount of oligomers (red line) and dimers (green line) are very close to those from experimental observations. More importantly, simulations seem to accurately reproduce the trends of the available data. They also match the qualitative knowledge for those species in the cases where no quantitative information was available (e.g. levels of Lamp2a, high molecular weight ASYN species, oligomeric species in proteasome). Specifically, levels of free Lamp2a seem to continuously decrease and reach zero levels on day 7. Interestingly, cell death is also observed in the laboratory experiments on day 7. As shown in Figure 7A, fluctuations in free Lamp2a levels indicate that the repression of CMA activity is not exclusively caused by the permanent binding of modified species to Lamp2a. It is also a result of the overload of the receptor caused either by the accumulated (due to overexpression and impaired degradation) unmodified monomers and dimers, or by the oligomers of un-modified ASYN. Therefore, two parallel processes for the repression of CMA activity evolve, one that gradually reduces the levels of free Lamp2a, and another that overuses the constantly declining levels of the free receptors. High molecular weight species of ASYN (higher than nonamers) remain at low levels (as expected from [8]), significantly below the levels of oligomers (trimers to nonamers). Levels of oligomeric species in the proteasome also appear to agree with the available experimental findings (ASYN species in proteasome represent only 0.5% of the total protein in the system as suggested in [18]).Simulation outputs did not accurately match the laboratory data in the case of WT ASYN monomers (Figure 7B). Nevertheless, the model’s behavior captured the trends of experimental observations and managed to reproduce the decrease of monomeric levels for the first few days and their subsequent rise after day 4. Considering that only the total levels of ASYN species in the cytoplasm could be experimentally measured, we chose (for the empirical-preparatory tuning and parameter estimation procedures) to give priority to the accurate simulation of dimeric and oligomeric ASYN, since these species are shown to induce the observed deregulations in neuronal homeostasis (see Background section). In contrast, the levels of monomeric ASYN are not significantly correlated with the pathogenic phenomena. In this sense, the lack of exact matching of their trends does not prevent us from valid qualitative conclusions from the model’s simulation results.

### Model predictions

The calibrated model was used as a testing platform for a number of hypothetical scenarios for which some experimental evidence was available either from the literature or from our lab. It is important to note that there was no update or prior calibration of the model with that experimental lab knowledge. Although there was no quantitative information available for any of the species in the model to compare with the simulation results, it was still possible to draw some conclusions in regards to the neuroprotective potential of these interventions. These were based on our knowledge that CMA suppression and the existence of soluble ASYN oligomers (which as described above are considered to be toxic) are responsible for reduced cell viability [8,14]. It is also important to note that all hypotheses were tested by changing the relevant parameters of the system and not any of the initial conditions. Thus, our in silico experiments aimed at intervening with the model of over-expressed ASYN system as this was structured and calibrated with the available experimental data. The long-term goal would be to unveil possible neuroprotective intervention strategies aiming at increasing cell viability in such ‘problematic’ systems.

### Non-toxic ASYN oligomer levels and decreased CMA inhibition are predicted when the ASYN production rate is reduced

**Figure 8:**
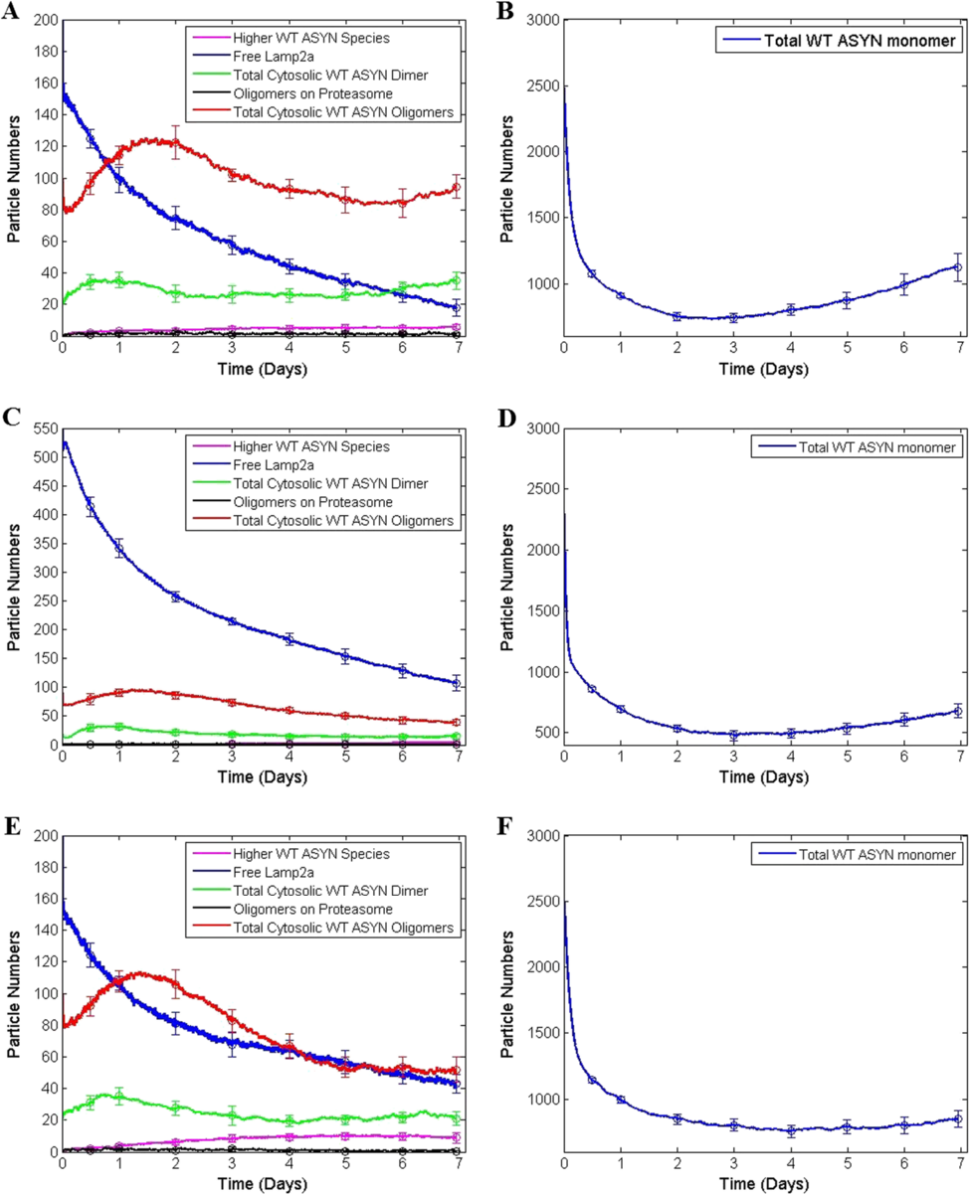
**Model predictions for three hypothetical scenarios for which no information was fed into the model during calibration.** In the first scenario the expression rate of ASYN was reduced by 50%, **(A)** levels of dimers (green) and oligomers (red) seem to return to their initial values after day 5 and free Lamp2a (blue) remains at higher levels at day 7 compared to Figure 7A. **(B)** This slightly increased concentration of Lamp2a has the opposite effect to the levels of WT ASYN monomers. **(C)** In a second hypothetical scenario, we tripled the initial amount of Lamp2a and ASYN oligomers and dimers appear in lower levels at day 7 than their initial amounts. Free Lamp2a on the other hand is increased. **(D)** WT alpha-synuclein monomers remain at lower levels for the last few days compared to those in the previous scenario. **(E)** In the third hypothetical scenario DA production was set to zero and at the end of the simulation levels of oligomers are reduced compared with their initial condition. Dimers return to their initial levels at day 7 while Lamp2a levels do not approach zero. **(F)** Levels of monomeric ASYN manifest a slower recovery trend compared to the other two scenarios.

In the first scenario tested, the production rate of ASYN was reduced by 50%. We aimed at checking whether the model recapitulates the association between WT ASYN over-expression and toxic phenomena and more specifically the existence of significant levels of oligomers and the over-occupation of Lamp2a receptors. As shown in Figure 8A, levels of dimers and oligomers are significantly lower compared to those in Figure 7A. In particular, during the last days of the experiment, where increased cell death is observed in vitro, these levels approach their initial values (day 0) where cell differentiation and protein overexpression begin. More specifically, we have recently shown that a significant difference in the number of intact nuclei between cells expressing endogenous ASYN and cells over-expressing ASYN starts to appear from the 5th day of culture [8]. On the other hand, the amount of free Lamp2a, despite being continuously reduced, it does not reach zero levels in the last day (as opposed to the case of Figure 7A). The levels of monomeric ASYN plotted in Figure 8B, are in general reduced compared to those in Figure 7B. This is most likely due to the slightly higher concentrations of free Lamp2a which aids the degradation of ASYN monomers as well as the lower production rate of ASYN. Thus, taking into account that oligomers remain at lower levels and there is no absolute consumption of Lamp2a, we can infer that the reduced production of ASYN has a significant effect on increasing cell survival probability (at least at day 7). This is in agreement with the correlation between ASYN over-expression and cytotoxicity observed experimentally [8].

### Tripling of Lamp2a levels is predicted to be sufficient to reduce the ASYN toxic effects on CMA

When we tripled the initial amount of Lamp2a receptors in the model (from 200 to 600) the model predicted that ASYN dimers and oligomers would be at lower levels at day 7 compared with those at the beginning of the simulation (see Figure 8C). Furthermore, the levels of free Lamp2a at day 7 appeared to be significantly higher than zero (a level at which CMA is expected to be completely inhibited) and reach levels similar to those at the beginning of the simulation where CMA is not inhibited. The levels of monomeric ASYN (Figure 8D) during the last few days are significantly lower compared to the initial conditions (Figure 7B). Moreover, the trend of ASYN monomers to recover during the last days of the simulation was abrogated. In conclusion, our model predicts an increased cell survival probability, and this agrees with experimental findings in our lab showing a higher turnover of ASYN species (monomeric as well as oligomeric) and significantly reduced ASYN-mediated neurotoxicity when Lamp2a is overexpressed. This was tested in three different experimental models: SH-SY5Y cells, primary rat cortical neurons and the living rat brain [37]. The in vivo data in particular are striking, in that the dopamine deficiency state was completely reversed, indicating that both the predictions of cellular and computational models are supported by the in vivo findings.

### Shutting off dopamine production leads to significantly reduced toxic phenomena mediated by ASYN oligomerization

It has been shown that inhibition of the DA production pathway in SH-SY5Y leads to reduced cell death and restoration of the lysosomal proteolytic mechanisms [14]. To test whether our model predictions are in agreement with such a behavior, we “shut off” DA production in the system by setting its production rate from SOURCE to 0. In order to simulate properly the experimental setup in which DA was shut off in non-dopamine deficient cells, the initial levels of DA were kept the same with those in the previous scenarios. The simulation outputs are presented in Figures 8E and F. Dimers again seem to return to their initial levels while oligomers appear in lower levels at the end of the simulation. In addition, levels of free Lamp2a receptors do not completely disappear at day 7. Monomeric ASYN species (Figure 8F) have significantly lower levels and when compared to the other two scenarios they manifest the smallest increasing trend during the last days of the simulation. An interesting feature of this hypothetical scenario, as shown in Figure 8E, is the increased levels of HMW ASYN species (line in magenta). Although at the end of the simulation the oligomers are significantly less, compared to those in the basic over-expression model, HMW species appear to remain in significant amounts. One possible explanation for this could be the exclusive oligomerization of ASYN by the non-modified pathway. Based on the hypotheses presented in [38,39] a more thorough explanation could be that the formation of HMW species can actually have a neuroprotective role by storing excessive protein which cannot be completely degraded via cell proteolytic mechanisms.

## Conclusions

We have presented a holistic biomolecular reactions model that successfully recapitulates the dynamic phenomena of ASYN overexpression and oligomerization and predicts the biological system’s behavior for a number of in-silico intervention scenarios not used for model calibration. The model allows for the first time, the estimation, at least in a qualitative manner, of the levels of the protein species that are capable of deregulating homeostasis. It also enables us to generate new hypotheses for intervention that could be tested experimentally.

A unique aspect of the model is that it describes the dynamics of protein modification by dopamine and its interaction with CMA, macroautophagy and proteasome pathways. The model has been validated by ASYN overexpression data from SH-SY5Y cells and predicted behaviors that indicate increased cell survival probability in three hypothesized intervention scenarios: halved production rate of ASYN, triplication of Lamp2a receptor levels in the lysosome, and cut-off of dopamine production. In all three cases the model predictions agreed with experimental lab results that were not presented to the model in advance. Obvious next steps in the analysis are the extension of the model to include cell death related pathways which incorporate the observed initiation of apoptosis, its inhibition and the resulting autophagic cell death [8,14]. Finally, the consideration of ASYN secretion and uptake from neighboring cells will allow us to test the different mechanisms proposed for disease propagation and in the long term to use an extended multi-scale version of the model as an in silico test-bed for investigating different theories.

## Methods

### Generation of stable cell lines, transfections and cell culture

The generation of the stable SH-SY5Y cell lines inducibly expressing WT alpha-synuclein (ASYN) was described previously in [8]. SH-SY5Y cells were cultured in RPMI 1640 medium containing 10% FBS, penicillin (100 U/ml), streptomycin (100 μg/ml), and 2 mM l-glutamine. Cells overexpressing either WT ASYN or β-galactosidase (bGAL, control cells) were cultured in the presence of 250 μg/ml G418 and 50 μg/ml hygromycin B. ASYN expression was switched off by the addition of doxycycline (0.5 μg/ml). Stock cultures were kept in the presence of doxycycline. Neuronal differentiation was performed with the addition of 10 μM all-trans retinoic acid for 6 d. This study did not use any animal or human subjects.

### Western immunoblotting

For extraction of cellular proteins, cells were harvested, washed twice with ice-cold PBS, and lysed with STEN lysis buffer (50 mm Tris, pH 7.6, 150 mm NaCl, 0.1% SDS, 1% NP-40, and 2 mm EDTA), plus a protease inhibitor cocktail (Roche) on ice for 20 min. Protein content was estimated using the Bradford method (Bio-Rad). Cells were processed for western blotting as previously described [16]. Denaturing gel electrophoresis was performed in 12% SDS-PAGE gels in Tris–glycine buffer. Immunoblotting was performed using the following antibodies: anti-ASYN (C20 rabbit polyclonal from Santa Cruz Biotechnology; or Syn-1 monoclonal antibody from BD Biosciences), anti-β-actin (mouse monoclonal; Sigma), anti Lamp2a (rabbit polyclonal, Abcam), anti-ERK (mouse monoclonal, Santa Cruz Biotechnology). All immunoblots were performed in triplicate. Quantification of bands on Western immunoblots was performed using Gel Analyzer software (Biosure). Differences in protein expression levels were quantified after standardization of all values using the appropriate loading controls (β-actin, ERK). All statistical analyses were performed using the Student’s t-test and p values of <0.05 were considered significant. All data are expressed as mean ± SE. A representative blot is shown in Additional file 3.

### Results quantification, data collection and processing

The results of Western Blot experiments (protein extract from 3 × 10^5^ cells, one measurement for every day of culture) were projected in Fuji films which were scanned by an EPSON Perfection 1200U scanner. The various bands identified in films were quantified in absolute intensity manner by the Gel Analyzer software. For the case of ASYN monomers (band identified at 17 kDa) and dimers (band identified at 35 kDa) it was possible to identify discrete bands which were measured independently. However, in the case of oligomers only diffused bands (smears) in the region of ~50 kDa to ~130 kDa could be seen and so, the whole smear band was quantified, to represent the sum of ASYN oligomers (trimmers to nonamers). Together with the protein extracts, three different known amounts of recombinant ASYN were co-measured (*rASYN*1 = 3.5*ng*, *rASYN*2 = 7*ng*, *rASYN*3 = 14*ng*) in order to quantify (by comparing the absolute intensities) the various bands shown in blots. It should be mentioned that since the modification of ASYN species could not be identified in Western blot context the quantification concerns the coupled levels of modified and un-modified ASYN species.

The procedure that was followed in order to extract the amount of monomer ASYN species in terms of particle numbers is described by the following equations:

(1) $$\text{mWTASYN}\left(\mathrm{ng}\right)=\frac{\left(\frac{\text{rASYN}1^{2}∙I_{m}\left(\text{ng}\right)}{I_{\text{rASYN}1}}+\frac{\text{rASYN}2^{2}∙I_{m}\left(\mathrm{ng}\right)}{I_{\text{rASYN}2}}+\frac{\text{rASYN}3^{2}∙I_{m}\left(\text{ng}\right)}{I_{\text{rASYN}3}}\right)}{3}$$

(2) $$\mathrm{mWTASYN}/\mathrm{cell}\left(\mathrm{ng}\right)=\frac{\mathrm{mWTASYN}\left(\mathrm{ng}\right)}{3*10^{5}}$$

(3) $$\mathrm{mWTASYN}/\mathrm{cell}\left(\mathrm{Par}.\mathrm{number}\right)=\frac{\mathrm{mWTASYN}/\mathrm{cell}\left(\mathrm{ng}\right)}{\mathrm{Mol}.\mathrm{weight}(\mathrm{mWTASYN}\left(\mathrm{ng}\right)}$$

where *mWTASYN* and *mWTASYN*/*cell* are the total and per cell weights (in *ng*) of the monomeric ASYN; *rASYN*1, *rASYN*2 and *rASYN*3 are the three weights in *ng* (3.5, 7 and 14 *ng*) of the recombinant monomeric ASYN used during data collection; *I*_*m*_ is the intensity of the monomeric ASYN and *I*_*rASYN*1_, *I*_*rASYN*2_ and *I*_*rASYN*3_ are the intensities of the three recombinant ASYN. Moreover, $\frac{\mathrm{mWTASYN}}{\mathrm{cell}}\left(\mathrm{Par}.\mathrm{number}\right)$ is the quantity of *WTASYN* per cell in particle numbers and it can be calculated by dividing the weight of monomeric ASYN per cell by the molecular weight of *mWTASYN* in *ng*. In order to convert the molecular weight of monomer ASYN from kDa (14.4 kDa) to *ng* we accepted that 1 Da = 1.660538921(73) × 10^-27^ kg.

It should be mentioned that in order to reduce the computational cost, but also to be able to simulate the model stochastically, we had to scale down the obtained particle numbers by a factor of 1000. This method has also been followed in [24] and by adopting it our scaled measurements resulted in similar levels for monomeric ASYN as in [24], thus allowing the direct comparison but also the utilization of various findings of this study (ratio of molecular species).

For the case of dimeric ASYN a similar procedure was followed, however the intensities of monomer ASYN were chosen as reference,

(4) $$\mathrm{dWTASYN}\left(\mathrm{ng}\right)=\frac{\mathrm{mWTASYN}*I_{d}\left(\mathrm{ng}\right)}{I_{m}}$$

(5) $$\mathrm{dWTASYN}/\mathrm{cell}\left(\mathrm{Par}.\mathrm{number}\right)=\frac{\frac{\mathrm{dWTASYN}\left(\mathrm{ng}\right)}{3*10^{5}}}{2*\mathrm{Mol}.\mathrm{weight}\left(\mathrm{mWTASYN}\left(\mathrm{ng}\right)\right)}$$

Unfortunately, as mentioned above, in the case of oligomers, a similar procedure could not be followed, since there was no direct intensity reference available. So, in order to obtain an approximation of the sum of particle numbers of oligomers, it was decided to compare the levels of the band representing the dimeric ASYN with the intensities of smear bands. In order to do so, these intensities were normalized based on the intensities of the three different amounts of recombinant ASYN. The ratio between the dimers and the oligomers of ASYN was then calculated, and the levels of oligomeric ASYN were calculated by multiplying the levels of dimeric ASYN by this ratio. Table 1 summarizes the particle numbers of ASYN species as calculated following the procedure described above.

**Table 1:** Quantitative experimental data

**Day**	**Particle number of monomeric WT ASYN**	**Particle number of dimeric WT ASYN**	**Sum of particle numbers of oligomeric WT ASYN**
**1**	2605	22	108
**2**	2106	18	139
**3**	1697	22	154
**4**	1277	33	140
**5**	1030	37	151
**6**	1318	54	153
**7**	1205	86	197

### Modeling and Simulation

The model structure was captured in CellDesigner (versions 4.1& 4.3) [31,32] in order to take advantage of the convenience of model definition in a graphical environment and exploit the strength of Systems Biology Graphical Notation SBGN [30]. Subsequently, the finalized model was transported (using the Systems Biology Workbench (SBW) [40]) to COPASI (version 4.10& 4.11) [41] in order to be mathematically defined (definition of rate laws and assignment of parameter values for reactions and initial amounts/particle numbers for species). COPASI was also employed for the simulation of the model where both deterministic (LSODA) and stochastic simulation algorithms [35] were used. The parameters of the simulation algorithms were chosen to have the default values set by the creators of the tool.

### Mathematical definition of the model and initial parameter values

The model developed consists of 136 reactions which involve 90 distinct biochemical species. It includes three (3) compartments (as described in Model development sub-section of Results and discussion) where the two of them (lysosomes and macroautophagy) are encapsulated inside the first (cytosol). In order to simplify the process and allow for stochastic simulations we assumed Mass Action kinetics. To account for the uncertainty of the experimental data used to validate the model and the intrinsic noise due to low molecular counts, we opted for stochastic rather than deterministic simulation methods.

To decrease the complexity of the model we used experimental knowledge (whenever available) to group similar reactions and assign the same rate constants, leading to a total number of 20 free kinetic parameters. Firstly, it was assumed that the oligomerization rates of WT ASYN and DA-modified ASYN in the cytoplasm were the same and that the only change caused by the modification was the inability of DA-modified species to form higher molecular weight complexes (aggregates). Furthermore (as suggested in [24]), the rates of formation of all oligomers were set to be the same except for the one for the dimers. The same approach was followed for the dissociation rates. These assumptions were also adopted for modeling the oligomerization process in the CMA compartment. In addition, in the CMA submodel the binding rate constants of Lamp2a and WT ASYN were grouped as follows: one constant for monomers and dimers and one for trimers to nonamers. The release rate was considered to be the same for all oligomers. It should be noted here that the modified protein rate constants regarding the CMA part of the model were different from those of unmodified forms. All parameters in the macroautophagy compartment were considered to be equal due to lack of experimental data suggesting that different protein species enter the autophagolysosome at different rates. For the same reason, the binding and degradation rates of all species were also grouped in the compartment modeling ASYN’s interaction with the proteasome.

The next step was to arrive at some initial approximations for the model parameters that would serve as starting points for model calibration. Many of them were taken from [24] and some others were extracted from other sources. To begin with, the production rate of WT ASYN and all oligomerization rates were set equal to those in [24]. As there was no information available about the production rate of DA, the modification rate of ASYN and the degradation of DA, we decided to use as initial values the production and removal rates of Reactive Oxygen Species (ROS) and the rate of modification they induce on WT ASYN from [24] in order to obtain initial values for the rates of these reactions in realistic boundaries. Both rates were revisited during the model calibration steps. In addition, for the CMA compartment of our model, we used the following rates from [24]: the binding rate of WT ASYN to Lamp2a, the internalization and Lamp2a releasing rates. The corresponding binding rates of DA-modified ASYN were increased by 20% based on findings in [15]. It was also decided that the internalization rates in the macroautophagy compartment would be approximated with the binding rates to Lamp2a. This decision was driven by the lack of detailed experimental data and the need to introduce realistic rates for this proteolytic machinery. However, this was only an initial estimate of the rate’s value and, as discussed in subsequent sections, it was adjusted using semi-quantitative data. Finally, the complex formation rate of ASYN species with the proteasome was also taken from [24]. Here, again, the rate was further adjusted computationally using semi-quantified but also qualitative experimental data.

All biomodel reactions and associated parameters are provided in Additional file 4: Table S1. This table also includes a description of all parameters and their groupings. Moreover, the aforementioned sources of initial parameter estimates are provided in Additional file 5: Table S1.

### Model calibration using experimental data

The model was calibrated exclusively with data produced in our lab. The experimental protocol regarding the over-expression of ASYN in SH-SY5Y cells was the same as in [8,14,16] thus allowing us to take advantage of experimental data collected at different times.

There were two types of available data. Semi-quantified relations (folds or percentages) to other species, or the same species in different experimental conditions, and concentration levels or particle numbers produced from experiments specifically designed for model calibration.

A step-by-step description of the model calibration process is shown in Figure 6. Once the model structure was finalized and initial parameter values were selected, the next step was to estimate more refined parameter values using model sub-units. Semi-quantified data, given in [14], and global optimization algorithm (Particle Swarm, for details see Parameter Estimation sub-section) were used to estimate the rate by which monomeric ASYN is inserted into the autophagosome compartment. It should be noted that because this parameter was estimated based on semi-quantified data and using a submodel in isolation of the rest of the system (the sub-model included only the reactions regarding the oligomerization process of WT ASYN and the autophagosome uptake of WT ASYN), we decided to adjust it again at the following step of empirical manual tuning.As shown in Figure 6, the next steps taken in order to calibrate the model with the available experimental data were: (a) the empirical-preparatory manual tuning, (b) the scaled sensitivity analysis of the model parameters, and (c) the estimation of significant parameters determined by sensitivity analysis using optimization algorithms. Each one of these steps is discussed in more detail in the following sub-sections.

### Empirical-preparatory tuning methodology

The set of parameters that were submitted for tuning in this step was decided based on empirical knowledge about their impact on the biological system’s behavior and also on the observed initial simulation results. The scope of this procedure was to manually tune a subset of the parameters in order to reach values that would drive the model to adequately converge to the qualitative knowledge for the biological system’s behavior, based on literature and experimental findings. These parameter values were used as initial guesses for the final parameter estimation step using global optimization algorithms. The simulation algorithm used in this phase was deterministic (LSODA in COPASI), but, in order to also take into account stochastic effects, a number of stochastic simulations were also run every time we acquired a good approximation of reality.

The model simulation outputs were reviewed based on the following qualitative criteria: (a) ASYN monomers present a sudden drop between day 1 and day 5 following the modification of the protein and then start recovering at day 6. (b) Dimers stay pretty much constant for the first 3 days, and then gradually increase until day 7 when they increase abruptly. (c) Oligomers (from trimers to nonamers) increase significantly from day 1 to day 2, continue to have a rising trend till day 6 and then increase acutely. (d) Levels of HMW species (above nonamers) are not significant, as indicated in [8]. (e) Finally, free Lamp2a levels gradually decrease approaching zero in the last days of the experiment, i.e., all receptors are occupied [14]. Suppression of Lamp2a is directly related to autophagic cell death initiation and thus it should only appear at later stages of the experiment in order to match laboratory observations. At each step of the empirical-preparatory tuning procedure of a parameter its value was adjusted following a trial and error procedure. The parameter values were first checked with respect to the ASYN monomer levels. If successful, it was then attempted to reproduce the desired behavior in terms of the levels of dimers, without affecting the previously matched levels of the monomers. At the last step of this procedure, the parameter values were adjusted, having in mind the behavior of the sum of oligomers. Levels and trends of Lamp2a and HMW species were also checked at each step for agreement with the targets set above.

### Sensitivity analysis

The sensitivity of the simulation results with respect to the model parameters was systematically analyzed to determine if the model was sufficiently robust and able to capture the true dynamic behavior of the biological system. Moreover, the results of sensitivity analysis, after empirically tuning the model, were used to identify the set of the most significant parameters that would be re-estimated using global optimization algorithms. This resulted in the reduction of the computational cost of the procedure. Setting all parameters and initial conditions as variables we calculated the scaled sensitivities with respect to the total amount of ASYN monomers, dimers and oligomers in the cytoplasm (e.g., all species for which quantitative experimental data was available). This was done by numerical differentiation using finite differences, a tool integrated in COPASI [41]. Scaled sensitivities were used since the previously mentioned species showed significant differences in their particle number levels. One should take particular note of the deterministic nature of the selected method and the fact that it only corresponds to local sensitivity analysis. This limitation was overcome by having obtained good estimates of parameter values prior to this step, derived by the empirical-preparatory manual tuning procedure. The sensitivity analysis results are summarized in Additional file 6: Table S1.

### Parameter estimation

Parameter estimation was the last step of the model calibration procedure. For this purpose, we used all available quantitative information as described above. The parameter set was chosen carefully after reviewing the scaled sensitivity analysis results. There were two categories of parameters to be estimated: rates of biochemical reactions and initial amount of species for which there was no available data.

For reactions rates, we initially used the values determined during the empirical-preparatory tuning step and a range for each parameter depending on our empirical knowledge on the suitability of the starting point. To reduce the computational cost and facilitate the good performance of the search algorithms, those intervals had to be constrained. This was done via an iterative optimization procedure where each margin was adjusted accordingly after a series of search runs. Additional file 5: Table S1 provides the estimated reaction rates, their initial values and used search margins.

The second category of parameters subjected to parameter estimation included the ratio of DA-modified and non-modified WT ASYN at the day of cells’ differentiation (day 0 of the simulation). This was possible for monomers and dimers of the protein by assuming that: the initial amount of modified monomers (dimers) is equal to the initial amount of the total monomers (dimers) minus the initial amount of the non-modified monomers (dimers). For this, we had to first estimate the initial amounts of modified and non-modified monomers and dimers.

It was not possible to follow a similar procedure for the modified and non-modified oligomeric species because there was no knowledge for each one of them separately (only the sum of them was known). To overcome this limitation, it was assumed that the levels of modified and non-modified oligomers were uniformly distributed across all oligomer sizes (trimers to nonamers).

Another set of parameters that needed to be estimated was the levels of HMW species of WT ASYN. Based on the conclusions made in [8] about the presence of HMW species in undetectable amounts within our cell system, we constrained their number to 15. This value was chosen based exclusively on empirical knowledge and was attempted to reflect the relative low levels of those species in comparison with the other forms of ASYN. Following a similar approach, we restricted the amount of oligomers bound to the proteasome to be less or equal to 20, taking into account that the amount of ASYN that relocates there, is approximately 0.5% of the total amount of the protein in the system [18].

All of the above information was inserted into the computational tool COPASI and in combination with the quantitative data shown in Table 1 was provided as input to Stochastic Global Optimization algorithms for parameter estimation. Stochastic algorithms are usually preferred over deterministic mainly because they offer the advantage of low computation cost and are most commonly used in parameter estimation problems for calibration of biomolecular reaction networks [42,43]. The four algorithms used in our study are: Genetic Algorithm (GA) [44], Genetic Algorithm with Stochastic Ranking (GASR) [45], Evolutionary Strategy algorithm (SRES) [44] and Particles Swarm algorithm (PS) [46]. The evaluation of the results was based firstly on the Objective Function and Root Mean Square Error and secondly on whether model simulations using the parameter values estimated by each algorithm, met the set criteria described in sub-section Empirical-preparatory Tuning Methodology.

The deterministic model simulation outputs using the parameters predicted by each algorithm are provided as supplementary material. As shown in Additional file 5: Figures S1 and S2, the GA failed to satisfy the requirements set for all species except from the HMW ASYN. Although GASR managed to capture the trends of oligomers and free Lamp2a, it gave less successful results with respect to the levels of dimers (Additional file 5: Figures S3 and S4). On the other hand, the SRES performed poorly in all cases (Additional file 5: Figures S5 and S6). Finally, we decided to accept the results of the PS algorithm (Additional file 5: Figures S7 and S8), which provided overall the best approximation of the expected system behavior and met all criteria set at the beginning of the process, although it ended up being the second best option in terms of RMSE and Objective function performance (behind the GASR). The final values of all parameters estimated using the PS algorithm are summarized in Additional file 5: Table S1.

It should be noted here that although it was attempted to find the best estimated values, this was not possible for all parameters due to the limited types of available data. For example, there was no data in relation to the species bound on Lamp2a at every time point, and as a result this rate could not be estimated directly. The same applies to species for which it was not possible to measure their levels. Thus, taking into account that the only available data were related to the amount of monomers, dimers and the sum of all oligomers of ASYN (restricted by the western blot method used to detect them), part of the system could not be directly parameterized.

### Model annotation

In order for the proposed computational model to be compliant with the MIRIAM guidelines (http://co.mbine.org/standards/miriam), a detailed model annotation procedure using the CellDesigner tool was followed, and was finalized for the pure SBML version of the model (Additional files 1 and 2) using the SBMLeditor tool [47]. MIRIAM qualifiers and MIRIAM URIs from Gene Ontology (GO), UniProt and Ontology of Chemical Entities of Biological Interest (CheBi) were used. Moreover, the model compartments, entities and reactions were annotated using Systems Biology Ontology (SBO) (http://www.ebi.ac.uk/sbo/) terms.

## Competing interests

The authors declare that they have no competing interests.

## Authors’ contributions

EO carried out the model development and calibration. ESM conceived and coordinated the theoretical study and provided input to model development and parameter estimation. DK participated on model development and calibration. MX and EE carried out wet-lab experiments. LS participated in the design of the experimental study. KV participated in the design of the experimental study, contributed to model curation and interpretation of the results. DK, EO and ESM wrote the manuscript. All authors read and approved the final manuscript.

## Supplementary Material

Additional file 1 **SBML code of the model including CellDesigner-specific annotations (SBGN information as given in Figures** 2, 3, 4** and **5**).** Click here for file

Additional file 2 **SBML code of the model (Pure SBML file).** The following SBML readers can be used to open and read the code: http://celldesigner.org/download.html or http://www.copasi.org/tiki-index.php?page=download. Click here for file

Additional file 3 Representative Western immunoblot. Click here for file

Additional file 4 **Model reactions and parameters.** A table listing the model’s biomolecular reactions categorized per model compartment along with their description and parameters. Click here for file

Additional file 5 **Simulation results of parameter estimation algorithms.** Figures of model outputs using different parameter values. Table providing initial and final parameter values as estimated by the chosen parameter estimation algorithm. Click here for file

Additional file 6 **Sensitivity analysis results.** A table summarizing the results of scaled sensitivity analysis as calculated in COPASI using the available experimental data. Click here for file
