# Supplementary figures and images for: In silico modeling of the effects of alpha-synuclein oligomerization on dopaminergic neuronal homeostasis
Source: BMC Syst Biol. 2014 May 13;8:54. doi: 10.1186/1752-0509-8-54 (PMC4062111; doi:10.1186/1752-0509-8-54)

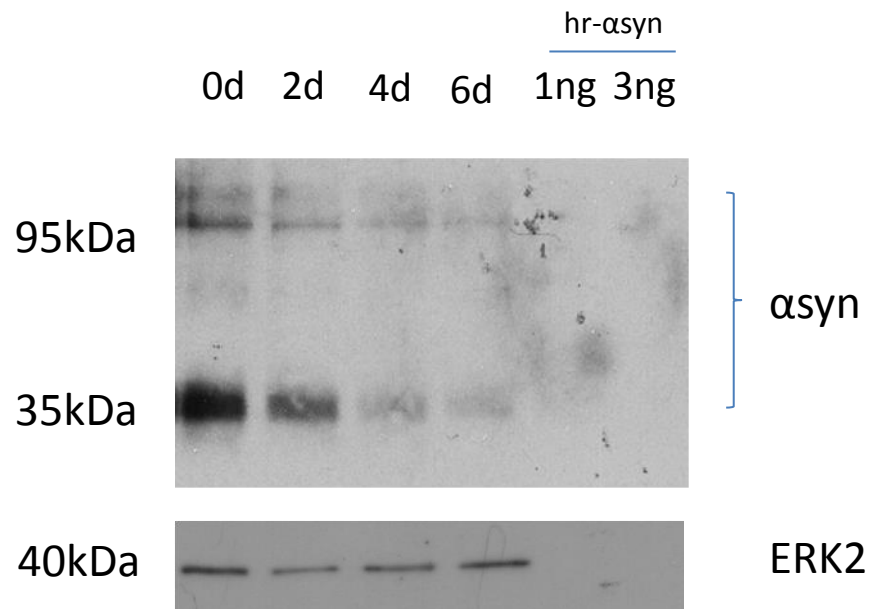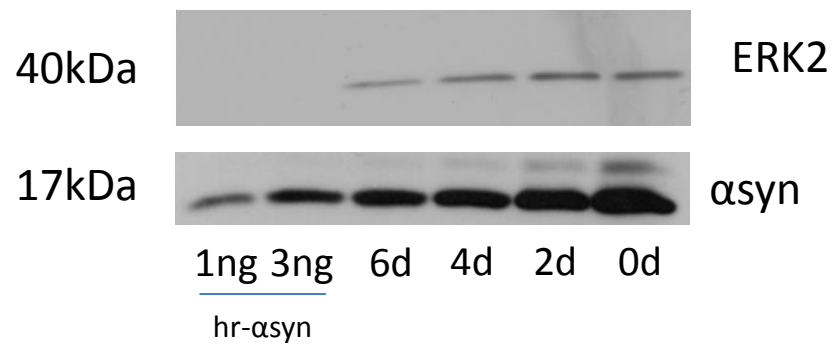

Supplement: Additional file 3 — Representative Western immunoblot. [file 1752-0509-8-54-S3.pdf]
