# Supplementary material for: In silico modeling of the effects of alpha-synuclein oligomerization on dopaminergic neuronal homeostasis
Source: BMC Syst Biol. 2014 May 13;8:54. doi: 10.1186/1752-0509-8-54 (PMC4062111; doi:10.1186/1752-0509-8-54)
Supplement: Additional file 4 — Model reactions and parameters. A table listing the model’s biomolecular reactions categorized per model compartment along with their description and parameters. [file 1752-0509-8-54-S4.pdf]

Table 1: List of biomolecular reactions and parameters categorized per model compartment. The last column provides the parameter groups as described in the section 'Model development'.

Description	Name	Reaction	Parameter	Parameter group
**ASYN & Dopamine production and modification**				
WT ASYN production	WTasynSynthesis	SOURCE -> WTasyn{Cytosol}	$k_{WTasynSynth}$	
Dopamine production	DopProduction	SOURCE -> Dopamine	$k_{DopProd}$	
WT ASYN modification from Dopamine	WTasynDopModification	WTasyn{Cytosol} + Dopamine -> DopModWTasyn{Cytosol}	$k_{WTasynDopMod}$	
Dopamine degradation	DopamineDegr	Dopamine -> Dopamine_degraded	$k_{DopDegr}$	
**ASYN oligomerization**				
WT ASYN Dimer formation	2merForm	2 * WTasyn{Cytosol} -> WTasyn2{Cytosol}	$k_{WTasyn2merForm}$	$k_{2merForm}$
Formation of modified ASYN Dimer	DopMod2merForm	2 * DopModWTasyn{Cytosol} -> DopModWTasyn2{Cytosol}	$k_{DopModWTasyn2merForm}$	$k_{2merForm}$
WT ASYN 3mer formation	3merForm	WTasyn2{Cytosol} + WTasyn{Cytosol} -> WTasyn3{Cytosol}	$k_{WTasyn3merForm}$	$k_{OligomerForm}$
WT ASYN 4mer formation	4merForm	WTasyn3{Cytosol} + WTasyn{Cytosol} -> WTasyn4{Cytosol}	$k_{WTasyn4merForm}$	$k_{OligomerForm}$
WT ASYN 5mer formation	5merForm	WTasyn4{Cytosol} + WTasyn{Cytosol} -> WTasyn5{Cytosol}	$k_{WTasyn5merForm}$	$k_{OligomerForm}$

WT ASYN 6mer formation	6merForm	WTasyn5{Cytosol} + WTasyn{Cytosol} -> WTasyn6{Cytosol}	$k_{WTasyn6merForm}$	$k_{OligomerForm}$
WT ASYN 7mer formation	7merForm	WTasyn6{Cytosol} + WTasyn{Cytosol} -> WTasyn7{Cytosol}	$k_{WTasyn7merForm}$	$k_{OligomerForm}$
WT ASYN 8mer formation	8merForm	WTasyn7{Cytosol} + WTasyn{Cytosol} -> WTasyn8{Cytosol}	$k_{WTasyn8merForm}$	$k_{OligomerForm}$
WT ASYN 9mer formation	9merForm	WTasyn8{Cytosol} + WTasyn{Cytosol} -> WTasyn9	$k_{WTasyn9merForm}$	$k_{OligomerForm}$
Formation of modified ASYN 3mer	DopMod3merForm	DopModWTasyn2{Cytosol} + DopModWTasyn{Cytosol} -> DopModWTasyn3{Cytosol}	$k_{DopModWTasyn3merForm}$	$k_{OligomerForm}$
Formation of modified ASYN 4mer	DopMod4merForm	DopModWTasyn3{Cytosol} + DopModWTasyn{Cytosol} -> DopModWTasyn4{Cytosol}	$k_{DopModWTasyn4merForm}$	$k_{OligomerForm}$
Formation of modified ASYN 5mer	DopMod5merForm	DopModWTasyn4{Cytosol} + DopModWTasyn{Cytosol} -> DopModWTasyn5{Cytosol}	$k_{DopModWTasyn5merForm}$	$k_{OligomerForm}$
Formation of modified ASYN 6mer	DopMod6merForm	DopModWTasyn5{Cytosol} + DopModWTasyn{Cytosol} -> DopModWTasyn6{Cytosol}	$k_{DopModWTasyn6merForm}$	$k_{OligomerForm}$
Formation of modified ASYN 7mer	DopMod7merForm	DopModWTasyn6{Cytosol} + DopModWTasyn{Cytosol} -> DopModWTasyn7{Cytosol}	$k_{DopModWTasyn7merForm}$	$k_{OligomerForm}$
Formation of modified ASYN	DopMod8merForm	DopModWTasyn7{Cytosol} +	$k_{DopModWTasyn8merForm}$	$k_{OligomerForm}$

8mer		DopModWTasyn{Cytosol} -> DopModWTasyn8{Cytosol}		
Formation of modified ASYN 9mer	DopMod9merForm	DopModWTasyn8{Cytosol} + DopModWTasyn{Cytosol} -> DopModWTasyn9	$k_{DopModWTasyn9merForm}$	$k_{OligomerForm}$
Aggregate Formation	AggregForm	WTasyn9 + WTasyn{Cytosol} -> HigherWTasynSPC + WTasynInHigher	$k_{AggrForm}$	$k_{OligomerForm}$
Aggregate growth	AggregGrowth	HigherWTasynSPC + WTasyn{Cytosol} -> HigherWTasynSPC + WTasynInHigher	$k_{AggrGrowth}$	
WT asyn Dimer disassociation	2merDis	WTasyn2{Cytosol} -> 2 * WTasyn{Cytosol}	$k_{WTasyn2merDis}$	$k_{DisRate}$
WT asyn 3mer disassociation	3merDis	WTasyn3{Cytosol} -> WTasyn2{Cytosol} + WTasyn{Cytosol}	$k_{WTasyn3merDis}$	$k_{DisRate}$
WT asyn 4mer disassociation	4merDis	WTasyn4{Cytosol} -> WTasyn3{Cytosol} + WTasyn{Cytosol}	$k_{WTasyn4merDis}$	$k_{DisRate}$
WT asyn 5mer disassociation	5merDis	WTasyn5{Cytosol} -> WTasyn4{Cytosol} + WTasyn{Cytosol}	$k_{WTasyn5merDis}$	$k_{DisRate}$
WT asyn 6mer disassociation	6merDis	WTasyn6{Cytosol} -> WTasyn5{Cytosol} + WTasyn{Cytosol}	$k_{WTasyn6merDis}$	$k_{DisRate}$
WT asyn 7mer disassociation	7merDis	WTasyn7{Cytosol} -> WTasyn6{Cytosol} + WTasyn{Cytosol}	$k_{WTasyn7merDis}$	$k_{DisRate}$
WT asyn 8mer disassociation	8merDis	WTasyn8{Cytosol} -> WTasyn7{Cytosol} + WTasyn{Cytosol}	$k_{WTasyn8merDis}$	$k_{DisRate}$

WT asyn 9mer disassociation	9merDis	WTasyn9 -> WTasyn8{Cytosol} + WTasyn{Cytosol}	$k_{WTasyn9merDis}$	$k_{DisRate}$
Disassociation of modified ASYN Dimer	DopMod2merDis	DopModWTasyn2{Cytosol} -> 2 * DopModWTasyn{Cytosol}	$k_{DopModWTasyn2merDis}$	$k_{DisRate}$
Disassociation of modified ASYN 3mer	DopMod3merDis	DopModWTasyn3{Cytosol} -> DopModWTasyn2{Cytosol} + DopModWTasyn{Cytosol}	$k_{DopModWTasyn3merDis}$	$k_{DisRate}$
Disassociation of modified ASYN 4mer	DopMod4merDis	DopModWTasyn4{Cytosol} -> DopModWTasyn3{Cytosol} + DopModWTasyn{Cytosol}	$k_{DopModWTasyn4merDis}$	$k_{DisRate}$
Disassociation of modified ASYN 5mer	DopMod5merDis	DopModWTasyn5{Cytosol} -> DopModWTasyn4{Cytosol} + DopModWTasyn{Cytosol}	$k_{DopModWTasyn5merDis}$	$k_{DisRate}$
Disassociation of modified ASYN 6mer	DopMod6merDis	DopModWTasyn6{Cytosol} -> DopModWTasyn5{Cytosol} + DopModWTasyn{Cytosol}	$k_{DopModWTasyn6merDis}$	$k_{DisRate}$
Disassociation of modified ASYN 7mer	DopMod7merDis	DopModWTasyn7{Cytosol} -> DopModWTasyn6{Cytosol} + DopModWTasyn{Cytosol}	$k_{DopModWTasyn7merDis}$	$k_{DisRate}$
Disassociation of modified ASYN 8mer	DopMod8merDis	DopModWTasyn8{Cytosol} -> DopModWTasyn7{Cytosol} + DopModWTasyn{Cytosol}	$k_{DopModWTasyn8merDis}$	$k_{DisRate}$
Disassociation of modified ASYN 9mer	DopMod9merDis	DopModWTasyn9 -> DopModWTasyn8{Cytosol} + DopModWTasyn{Cytosol}	$k_{DopModWTasyn9merDis}$	$k_{DisRate}$

**CMA component**				
Binding of WT ASYN Monomer with Lamp2a	WTasynLampBind	WTasyn{Cytosol} + Lamp2a -> WTasyndegr	$k_{WTasynLampBind}$	$k_{WTasyn1\_2merBindOnLamp}$
Binding of WT ASYN Dimer with Lamp2a	WTasyn2LampBind	WTasyn2{Cytosol} + Lamp2a -> WTasyn2degr	$k_{WTasyn2merLampBind}$	$k_{WTasyn1\_2merBindOnLamp}$
Binding of WT ASYN 3mer on Lamp2a	WTasyn3merBindOnLamp	Lamp2a + WTasyn3{Cytosol} -> WTasyn3merOnLamp	$k_{WTasynr3merLampBind}$	$k_{WTOligoBindOnLamp}$
Binding of WT ASYN 4mer on Lamp2a	WTasyn4merBindOnLamp	Lamp2a + WTasyn4{Cytosol} -> WTasyn4merOnLamp	$k_{WTasynr4merLampBind}$	$k_{WTOligoBindOnLamp}$
Binding of WT ASYN 5mer with Lamp2a	WTasyn5merBindOnLamp	WTasyn5{Cytosol} + Lamp2a -> WTasyn5merOnLamp	$k_{WTasynr5merLampBind}$	$k_{WTOligoBindOnLamp}$
Binding of WT ASYN 6mer on Lamp2a	WTasyn6merBindOnLamp	Lamp2a + WTasyn6{Cytosol} -> WTasyn6merOnLamp	$k_{WTasynr6merLampBind}$	$k_{WTOligoBindOnLamp}$
Binding of WT ASYN 7mer with Lamp2a	WTasyn7merBindOnLamp	WTasyn7{Cytosol} + Lamp2a -> WTasyn7merOnLamp	$k_{WTasynr7merLampBind}$	$k_{WTOligoBindOnLamp}$
Binding of WT ASYN 8mer on Lamp2a	WTasyn8merBindOnLamp	WTasyn8merOnLamp + WTasyn8{Cytosol} -> Lamp2a	$k_{WTasynr8merLampBind}$	$k_{WTOligoBindOnLamp}$
Binding of WT ASYN 9mer with Lamp2a	WTasyn9merBindOnLamp	WTasyn9 + Lamp2a -> WTasyn9merOnLamp	$k_{WTasynr9merLampBind}$	$k_{WTOligoBindOnLamp}$
WT ASYN Monomer uptake from lysosome	WTasynLysosUptake	WTasyndegr -> Lamp2a + WTasyn{Lysosome}	$k_{WTasynLysUptk}$	
WT ASYN Dimer uptake from lysosome	WTasyn2LysosUptake	WTasyn2degr -> Lamp2a + WTasyn2{Lysosome}	$k_{WTasyn2LysUptk}$	

WT ASYN Monomer degradation in the lysosome	WTasyn1merCMADegr	WTasyn{Lysosome} -> WTasynCMADegr	$k_{WTasynLysDegr}$	
WT ASYN Dimer degradation in the lysosome	WTasyn2merCMADegr	WTasyn2{Lysosome} -> WTasyn2merCMADegr	$k_{WTasyn2merLysDegr}$	
Release of WT ASYN 3mer from lamp2a	LampFree3merWT	WTasyn3merOnLamp -> WTasyn3{Cytosol} + Lamp2a	$k_{WTasyn3merLampFree}$	$k_{LampFreeWTasyn}$
Release of WT ASYN 4mer from lamp2a	LampFree4merWT	WTasyn4merOnLamp -> WTasyn4{Cytosol} + Lamp2a	$k_{WTasyn4merLampFree}$	$k_{LampFreeWTasyn}$
Release of WT ASYN 5mer from lamp2a	LampFree5merWT	WTasyn5merOnLamp -> WTasyn5{Cytosol} + Lamp2a	$k_{WTasyn5merLampFree}$	$k_{LampFreeWTasyn}$
Release of WT ASYN 6mer from lamp2a	LampFree6merWT	WTasyn6merOnLamp -> WTasyn6{Cytosol} + Lamp2a	$k_{WTasyn6merLampFree}$	$k_{LampFreeWTasyn}$
Release of WT ASYN 7mer from lamp2a	LampFree7merWT	WTasyn7merOnLamp -> WTasyn7{Cytosol} + Lamp2a	$k_{WTasyn7merLampFree}$	$k_{LampFreeWTasyn}$
Release of WT ASYN 8mer from lamp2a	LampFree8merWT	WTasyn8merOnLamp -> WTasyn8{Cytosol} + Lamp2a	$k_{WTasyn8merLampFree}$	$k_{LampFreeWTasyn}$
Release of WT ASYN 9mer from lamp2a	LampFree9merWT	WTasyn9merOnLamp -> WTasyn9 + Lamp2a	$k_{WTasyn9merLampFree}$	$k_{LampFreeWTasyn}$
Formation of WT ASYN Dimer on Lamp2a	WTasyn2merFormOnLamp	WTasyn{Cytosol} + WTasyndegr -> WTasyn2degr	$k_{WTasyn2merLampForm}$	$k_{2merForm}$
Formation of WT ASYN 3mer on Lamp2a	WTasyn3merFormOnLamp	WTasyn{Cytosol} + WTasyn2merOnLamp -> WTasyn3merOnLamp	$k_{WTasyn3merLampForm}$	$k_{OligomerForm}$

Formation of WT ASYN 4mer on Lamp2a	WTasyn4merFormOnLamp	WTasyn{Cytosol} + WTasyn3merOnLamp -> WTasyn4merOnLamp	$k_{WTasyn4merLampForm}$	$k_{OligomerForm}$
Formation of WT ASYN 5mer on Lamp2a	WTasyn5merFormOnLamp	WTasyn{Cytosol} + WTasyn4merOnLamp -> WTasyn5merOnLamp	$k_{WTasyn5merLampForm}$	$k_{OligomerForm}$
Formation of WT ASYN 6mer on Lamp2a	WTasyn6merFormOnLamp	WTasyn5merOnLamp + WTasyn{Cytosol} -> WTasyn6merOnLamp	$k_{WTasyn6merLampForm}$	$k_{OligomerForm}$
Formation of WT ASYN 7mer on Lamp2a	WTasyn7merFormOnLamp	WTasyn6merOnLamp + WTasyn{Cytosol} -> WTasyn7merOnLamp	$k_{WTasyn7merLampForm}$	$k_{OligomerForm}$
Formation of WT ASYN 8mer on Lamp2a	WTasyn8merFormOnLamp	WTasyn{Cytosol} + WTasyn7merOnLamp -> WTasyn8merOnLamp	$k_{WTasyn8merLampForm}$	$k_{OligomerForm}$
Formation of WT ASYN 9mer on Lamp2a	WTasyn9merFormOnLamp	WTasyn{Cytosol} + WTasyn8merOnLamp -> WTasyn9merOnLamp	$k_{WTasyn9merLampForm}$	$k_{OligomerForm}$
Binding of modified WT ASYN on Lamp2a and inhibition of it	DopModWTasynCMAInhibition	Lamp2a + DopModWTasyn{Cytosol} -> DopModWTasynOnLamp	$k_{DopModWTasynLampBind}$	
Formation of modified ASYN Dimer on Lamp2a	DopWTasyn2merFormOnLamp	WTasyn{Cytosol} + WTasyndegr -> WTasyn2degr	$k_{DopModWTasyn2merFormLamp}$	$k_{2merForm}$
Formation of modified WT ASYN 3mer on Lamp2a	DopWTasyn3merFormOnLamp	WTasyn{Cytosol} + WTasyn2degr -> WTasyn3merOnLamp	$k_{DopModWTasyn3merFormLamp}$	$k_{OligomerForm}$

Formation of modified WT ASYN 4mer on Lamp2a	DopWTasyn4merFormOnLamp	WTasyn{Cytosol} + WTasyn3merOnLamp -> WTasyn4merOnLamp	$k_{DopModWTasyn4merFormLamp}$	$k_{OligomerForm}$
Formation of modified WT ASYN 5mer on Lamp2a	DopWTasyn5merFormOnLamp	WTasyn{Cytosol} + WTasyn4merOnLamp -> WTasyn5merOnLamp	$k_{DopModWTasyn5merFormLamp}$	$k_{OligomerForm}$
Formation of modified WT ASYN 6mer on Lamp2a	DopWTasyn6merFormOnLamp	WTasyn5merOnLamp + WTasyn{Cytosol} -> WTasyn6merOnLamp	$k_{DopModWTasyn6merFormLamp}$	$k_{OligomerForm}$
Formation of modified ASYN 7mer on Lamp2a	DopWTasyn7merFormOnLamp	WTasyn6merOnLamp + WTasyn{Cytosol} -> WTasyn7merOnLamp	$k_{DopModWTasyn7merFormLamp}$	$k_{OligomerForm}$
Formation of modified ASYN 8mer on Lamp2a	DopWTasyn8merFormOnLamp	WTasyn{Cytosol} + WTasyn7merOnLamp -> WTasyn8merOnLamp	$k_{DopModWTasyn8merFormLamp}$	$k_{OligomerForm}$
Formation of modified ASYN 9mer on Lamp2a	WTasyn9merFormOnLamp	WTasyn{Cytosol} + WTasyn8merOnLamp -> WTasyn9merOnLamp	$k_{DopModWTasyn9merFormLamp}$	$k_{OligomerForm}$
**Macroautophagy component**				
WT ASYN Monomer uptake from the autophagosome	AutophagosomeUptakeWTasyn	WTasyn{Cytosol} -> WTasyn{M/autophagy&OtherLysDegrPath}	$k_{WTasynAutophUptk}$	$k_{OligAutophagUptake}$
WT ASYN Dimer uptake from the autophagosome	AutophagosomeUptakeWTasyn2mer	WTasyn2{Cytosol} -> WTasyn2{M/autophagy&OtherLysDegrPath}	$k_{WTasyn2merAutophUptk}$	$k_{OligAutophagUptake}$

WT ASYN 3mer uptake from the autophagosome	AutophagosomeUptakeWTasyn3mer	WTasyn3{Cytosol} -> WTasyn3{M/autophagy&OtherLysDegrPath}	$k_{WTasyn3merAutophUptk}$	$k_{OligAutophagUptake}$
WT ASYN 4mer uptake from the autophagosome	AutophagosomeUptakeWTasyn4mer	WTasyn4{Cytosol} -> WTasyn4{M/autophagy&OtherLysDegrPath}	$k_{WTasyn4merAutophUptk}$	$k_{OligAutophagUptake}$
WT ASYN 5mer uptake from the autophagosome	AutophagosomeUptakeWTasyn5mer	WTasyn5{Cytosol} -> WTasyn5{M/autophagy&OtherLysDegrPath}	$k_{WTasyn5merAutophUptk}$	$k_{OligAutophagUptake}$
WT ASYN 6mer uptake from the autophagosome	AutophagosomeUptakeWTasyn6mer	WTasyn6{Cytosol} -> WTasyn6{M/autophagy&OtherLysDegrPath}	$k_{WTasyn6merAutophUptk}$	$k_{OligAutophagUptake}$
WT ASYN 7mer uptake from the autophagosome	AutophagosomeUptakeWTasyn7mer	WTasyn7{Cytosol} -> WTasyn7{M/autophagy&OtherLysDegrPath}	$k_{WTasyn7merAutophUptk}$	$k_{OligAutophagUptake}$
WT ASYN 8mer uptake from the autophagosome	AutophagosomeUptakeWTasyn8mer	WTasyn8{Cytosol} -> WTasyn8{M/autophagy&OtherLysDegrPath}	$k_{WTasyn8merAutophUptk}$	$k_{OligAutophagUptake}$
Modified WT ASYN Monomer uptake from the autophagosome	DopModAutophagosomeUptake	DopModWTasyn{Cytosol} -> DopModWTasyn{M/autophagy&OtherLysDegrPath}	$k_{DopModAutophUptk}$	$k_{OligAutophagUptake}$
Modified WT ASYN Dimer uptake from the autophagosome	DopModAutophagosomeUptake2mer	DopModWTasyn2{Cytosol} -> DopModWTasyn2{M/autophagy&OtherLysDegrPath}	$k_{DopMod2merAutophUptk}$	$k_{OligAutophagUptake}$
Modified WT ASYN 3mer	DopModAutophagosomeU	DopModWTasyn3{Cytosol} ->	$k_{DopMod3merAutophUptk}$	$k_{OligAutophagUptake}$

uptake from the autophagosome	ptake3mer	DopModWTasyn3{M/autophagy&OtherLysDegrPath}		
Modified WT ASYN 4mer uptake from the autophagosome	DopModAutophagosomeUptake4mer	DopModWTasyn4{Cytosol} -> DopModWTasyn4{M/autophagy&OtherLysDegrPath}	$k_{DopMod4merAutophUptk}$	$k_{OligAutophagUptake}$
Modified WT ASYN 5mer uptake from the autophagosome	DopModAutophagosomeUptake5mer	DopModWTasyn5{Cytosol} -> DopModWTasyn5{M/autophagy&OtherLysDegrPath}	$k_{DopMod5merAutophUptk}$	$k_{OligAutophagUptake}$
Modified WT ASYN 6mer uptake from the autophagosome	DopModAutophagosomeUptake6mer	DopModWTasyn6{Cytosol} -> DopModWTasyn6{M/autophagy&OtherLysDegrPath}	$k_{DopMod6merAutophUptk}$	$k_{OligAutophagUptake}$
Modified WT ASYN 7mer uptake from the autophagosome	DopModAutophagosomeUptake7mer	DopModWTasyn7{Cytosol} -> DopModWTasyn7{M/autophagy&OtherLysDegrPath}	$k_{DopMod7merAutophUptk}$	$k_{OligAutophagUptake}$
Modified WT ASYN 8mer uptake from the autophagosome	DopModAutophagosomeUptake8mer	DopModWTasyn8{Cytosol} -> DopModWTasyn8{M/autophagy&OtherLysDegrPath}	$k_{DopMod8merAutophUptk}$	$k_{OligAutophagUptake}$
Degradation of WT ASYN monomer from macroautophagy	M/autophagyWTasyn1Degr	WTasyn{M/autophagy&OtherLysDegrPath} -> WTasynmerM/Adegr	$k_{WTasynM/ADegr}$	$k_{M/autophagyDegr}$
WT ASYN Dimer degradation by macroautophagy	M/autophagyWTasyn2Degr	WTasyn2{M/autophagy&OtherLysDegrPath} -> WTasyn2merM/Adegr	$k_{WTasyn2merM/ADegr}$	$k_{M/autophagyDegr}$
WT ASYN 3mer degradation by macroautophagy	M/autophagyWTasyn3Degr	WTasyn3{M/autophagy&OtherLysDegrPath} -> WTasyn3merM/Adegr	$k_{WTasyn3merM/ADegr}$	$k_{M/autophagyDegr}$

WT ASYN 4mer degradation by macroautophagy	M/autophagyWTasyn4Degr	WTasyn4{M/autophagy&OtherLysDegrPath} -> WTasyn4merM/Adegrr	$k_{WTasyn4merM/ADegr}$	$k_{M/autophagyDegr}$
WT ASYN 5mer degradation by macroautophagy	M/autophagyWTasyn5Degr	WTasyn5{M/autophagy&OtherLysDegrPath} -> WTasyn5merM/Adegr	$k_{WTasyn5merM/ADegr}$	$k_{M/autophagyDegr}$
WT ASYN 6mer degradation by macroautophagy	M/autophagyWTasyn6Degr	WTasyn6{M/autophagy&OtherLysDegrPath} -> WTasyn6merM/Adegr	$k_{WTasyn6merM/ADegr}$	$k_{M/autophagyDegr}$
WT ASYN 7mer degradation by macroautophagy	M/autophagyWTasyn7Degr	WTasyn7{M/autophagy&OtherLysDegrPath} -> WTasyn7merM/Adegr	$k_{WTasyn7merM/ADegr}$	$k_{M/autophagyDegr}$
WT ASYN 8mer degradation by macroautophagy	M/autophagyWTasyn8Degr	WTasyn8{M/autophagy&OtherLysDegrPath} -> WTasyn8merM/Adegr	$k_{WTasyn8merM/ADegr}$	$k_{M/autophagyDegr}$
Degradation of modified WT ASYN monomer from macroautophagy	M/autophagyDopModWTasyn1Degr	DopModWTasyn{M/autophagy&OtherLysDegrPath} -> DopModWTasyn1merM/Adegr	$k_{DopModM/ADegr}$	$k_{M/autophagyDegr}$
Degradation of modified WT ASYN Dimer from macroautophagy	M/autophagyDopModWTasyn2Degr	DopModWTasyn2{M/autophagy&OtherLysDegrPath} -> DopModWTasyn2merM/Adegr	$k_{DopMod2merM/ADegr}$	$k_{M/autophagyDegr}$
Degradation of modified WT ASYN 3mer from macroautophagy	M/autophagyDopModWTasyn3Degr	DopModWTasyn3{M/autophagy&OtherLysDegrPath} -> DopModWTasyn3merM/Adegr	$k_{DopMod3merM/ADegr}$	$k_{M/autophagyDegr}$
Degradation of modified WT ASYN 4mer from macroautophagy	M/autophagyDopModWTasyn4Degr	DopModWTasyn4{M/autophagy&OtherLysDegrPath} -> DopModWTasyn4merM/Adegr	$k_{DopMod4merM/ADegr}$	$k_{M/autophagyDegr}$
Degradation of modified WT ASYN 5mer from	M/autophagyDopModWTasyn5Degr	DopModWTasyn5{M/autophagy&OtherLysDegrPath} ->	$k_{DopMod5merM/ADegr}$	$k_{M/autophagyDegr}$

macroautophagy		DopModWTasyn6merM/Adegr		
Degradation of modified WT ASYN 6mer from macroautophagy	M/autophagyDopModWTasyn6Degr	DopModWTasyn6{M/autophagy&OtherLysDegrPath} -> DopModWTasyn7merM/Adegr	$k_{DopMod6merM/ADegr}$	$k_{M/autophagyDegr}$
Degradation of modified WT ASYN 7mer from macroautophagy	M/autophagyDopModWTasyn7Degr	DopModWTasyn7{M/autophagy&OtherLysDegrPath} -> DopModWTasyn7merM/Adegr	$k_{DopMod7merM/ADegr}$	$k_{M/autophagyDegr}$
Degradation of modified WT ASYN 8mer from macroautophagy	M/autophagyDopModWTasyn8Degr	DopModWTasyn8{M/autophagy&OtherLysDegrPath} -> DopModWTasyn8merM/Adegr	$k_{DopMod8merM/ADegr}$	$k_{M/autophagyDegr}$
**Proteasome component**				
Binding of WT ASYN 3mer with the proteasome	3merProtBind	WTasyn3{Cytosol} + Proteasome -> ProtWTasyn3	$k_{WTasyn3merProtBind}$	$k_{ProteasomeBind}$
Binding of WT ASYN 4mer with the proteasome	4merProtBind	WTasyn4{Cytosol} + Proteasome -> ProtWTasyn4	$k_{WTasyn4merProtBind}$	$k_{ProteasomeBind}$
Binding of WT ASYN 5mer with the proteasome	5merProtBind	WTasyn5{Cytosol} + Proteasome -> ProtWTasyn5	$k_{WTasyn5merProtBind}$	$k_{ProteasomeBind}$
Binding of WT ASYN 6mer with the proteasome	6merProtBind	WTasyn6{Cytosol} + Proteasome -> ProtWTasyn6	$k_{WTasyn6merProtBind}$	$k_{ProteasomeBind}$
Binding of WT ASYN 7mer with the proteasome	7merProtBind	WTasyn7{Cytosol} + Proteasome -> ProtWTasyn7	$k_{WTasyn7merProtBind}$	$k_{ProteasomeBind}$
Binding of WT ASYN 8mer with the proteasome	8merProtBind	WTasyn8{Cytosol} + Proteasome -> ProtWTasyn8	$k_{WTasyn8merProtBind}$	$k_{ProteasomeBind}$

Binding of WT ASYN 9mer with the proteasome	9merProtBind	WTasyn9 + Proteasome -> ProtWTasyn9	$k_{WTasyn9merProtBind}$	$k_{ProteasomeBind}$
Binding of modified WT ASYN 3mer with the proteasome	DopMod3merProtBind	DopModWTasyn3{Cytosol} + Proteasome -> ProtDopModWTasyn3	$k_{DopMod3merProtBind}$	$k_{ProteasomeBind}$
Binding of modified WT ASYN 4mer with the proteasome	DopMod4merProtBind	DopModWTasyn4{Cytosol} + Proteasome -> ProtDopModWTasyn4	$k_{DopMod4merProtBind}$	$k_{ProteasomeBind}$
Binding of modified WT ASYN 5mer with the proteasome	DopMod5merProtBind	DopModWTasyn5{Cytosol} + Proteasome -> ProtDopModWTasyn5	$k_{DopMod5merProtBind}$	$k_{ProteasomeBind}$
Binding of modified WT ASYN 6mer with the proteasome	DopMod6merProtBind	DopModWTasyn6{Cytosol} + Proteasome -> ProtDopModWTasyn6	$k_{DopMod6merProtBind}$	$k_{ProteasomeBind}$
Binding of modified WT ASYN 7mer with the proteasome	DopMod7merProtBind	DopModWTasyn7{Cytosol} + Proteasome -> ProtDopModWTasyn7	$k_{DopMod7merProtBind}$	$k_{ProteasomeBind}$
Binding of modified WT ASYN 8mer with the proteasome	DopMod8merProtBind	DopModWTasyn8{Cytosol} + Proteasome -> ProtDopModWTasyn8	$k_{DopMod8merProtBind}$	$k_{ProteasomeBind}$
Binding of modified WT ASYN 9mer with the proteasome	DopMod9merProtBind	DopModWTasyn9 + Proteasome -> ProtDopModWTasyn9	$k_{DopMod9merProtBind}$	$k_{ProteasomeBind}$
Binding of HMW species with the proteasome	WTasynHigherSPCprotInh	HigherWTasynSPC + Proteasome -> ProtWTasynHigherSPC	$k_{WTasynHighProtBind}$	$k_{ProteasomeBind}$
Degradation of WT ASYN 3mer from the proteasome	ProtFree3merWT	ProtWTasyn3 -> Proteasome	$k_{WTasyn3merProtFree}$	$k_{ProtOligDegr}$
Degradation of WT ASYN 4mer from the proteasome	ProtFree4merWT	ProtWTasyn4 -> Proteasome	$k_{WTasyn4merProtFree}$	$k_{ProtOligDegr}$
Degradation of WT ASYN 5mer	ProtFree5merWT	ProtWTasyn5 -> Proteasome	$k_{WTasyn5merProtFree}$	$k_{ProtOligDegr}$

from the proteasome				
Degradation of WT ASYN 6mer from the proteasome	ProtFree6merWT	ProtWTasyn6 -> Proteasome	$k_{WTasyn6merProtFree}$	$k_{ProtOligDegr}$
Degradation of WT ASYN 7mer from the proteasome	ProtFree7merWT	ProtWTasyn7 -> Proteasome	$k_{WTasyn7merProtFree}$	$k_{ProtOligDegr}$
Degradation of WT ASYN 8mer from the proteasome	ProtFree8merWT	ProtWTasyn8 -> Proteasome	$k_{WTasyn8merProtFree}$	$k_{ProtOligDegr}$
Degradation of WT ASYN 9mer from the proteasome	ProtFree9merWT	ProtWTasyn9 -> Proteasome	$k_{WTasyn9merProtFree}$	$k_{ProtOligDegr}$
Degradation of modified WT ASYN 3mer from the proteasome	ProtFree3merDopWT	ProtDopModWTasyn3 -> Proteasome	$k_{DopMod3merProtFree}$	$k_{ProtOligDegr}$
Degradation of modified WT ASYN 4mer from the proteasome	ProtFree4merDopWT	ProtDopModWTasyn4 -> Proteasome	$k_{DopMod4merProtFree}$	$k_{ProtOligDegr}$
Degradation of modified WT ASYN 5mer from the proteasome	ProtFree5merDopWT	ProtDopModWTasyn5 -> Proteasome	$k_{DopMod5merProtFree}$	$k_{ProtOligDegr}$
Degradation of modified WT ASYN 6mer from the proteasome	ProtFree6merDopWT	ProtDopModWTasyn6 -> Proteasome	$k_{DopMod6merProtFree}$	$k_{ProtOligDegr}$
Degradation of modified WT ASYN 7mer from the proteasome	ProtFree7merDopWT	ProtDopModWTasyn7 -> Proteasome	$k_{DopMod7merProtFree}$	$k_{ProtOligDegr}$

Degradation of modified WT ASYN 8mer from the proteasome	ProtFree8merDopWT	ProtDopModWTasyn8 -> Proteasome	$k_{DopMod8merProtFree}$	$k_{ProtOligDegr}$
Degradation of modified WT ASYN 9mer from the proteasome	ProtFree9merDopWT	ProtDopModWTasyn9 -> Proteasome	$k_{DopMod9merProtFree}$	$k_{ProtOligDegr}$
