# Supplementary material for: In silico modeling of the effects of alpha-synuclein oligomerization on dopaminergic neuronal homeostasis
Source: BMC Syst Biol. 2014 May 13;8:54. doi: 10.1186/1752-0509-8-54 (PMC4062111; doi:10.1186/1752-0509-8-54)
Supplement: Additional file 5 — Simulation results of parameter estimation algorithms. Figures of model outputs using different parameter values. Table providing initial and final parameter values as estimated by the chosen parameter estimation algorithm. [file 1752-0509-8-54-S5.pdf]

Table 1: Initial and final values of kinetic parameters estimated by the Particles Swarm parameter estimation algorithm in COPASI

Parameter Name	Reference Values (starting point for Manual Empirical-preparatory Tuning)	Initial Value for parameter estimation (after Manual Empirical-preparatory Tuning)	Margins	Estimated value (by global stochastic optimization)
$k_{WTasyn2merForm}$	5e-12 [1]	6.89778e-10	[6.89778e-12 , 6.89778e-08]	1.46294e-09
$k_{OligomerForm}$	5e-10 [1]	8.1732e-09	[8.1732e-11 , 8.1732e-07]	3.3505e-08
$k_{ProteasomeBind}$	1.7e-09 [1]	1.7e-09	[1.7e-11 , 1.7e-07]	3.42469e-09
$k_{OligAutophagUptake}$	4e-08 [1]	2.38913e-06	[2.38913e-08 , 2.38913e-04]	2.39034e-08
$k_{LampFreeWTasyn}$	0.001 [1]	0.001	[0.0001 , 0.01]	0.000304457
$k_{ProtOligDegr}$	1e-02 [1]	3.70096e-05	[3.70096e-06 , 3.70096e-04]	0.000370096
$k_{WTasyn1\_2merBindOnLamp}$	4e-08 [1]	4e-08	[4e-10 , 4e-06]	6.86546e-07
$k_{WTOligoBindOnLamp}$	4e-08 [1]	4e-08	[4e-10 , 4e-06]	4e-06
$k_{DisRate}$	5e-09 [1]	5e-09	[5e-11 , 5e-07]	4.99953e-07
$k_{DopModWTasynLampBind}$	4.8e-08 [1,2]	8.268e-08	[8.268e-10 , 8.268e-06]	7.6716e-09
$k_{WTasynSynth}$	2.8e-02 [1]	0.011908	[0.0011908 , 0.11908]	0.0294219
$k_{DopProd}$	2.0e-05 [1]	0.01	[0.001 , 0.1]	0.0791823
$k_{WTasynDopMod}$	5e-08 [1]	5e-08	[5e-10 , 5e-10]	6.74768e-07
$k_{DopDegr}$	0.001 [1]	0.001	[0.0001 , 0.01]	0.00679501
$k_{AggrGrowth}$	5e-09 [1]	5e-09	[5e-11 , 5e-07]	4.90556e-07
$k_{WTasynLysUptk}$	0.001 [1]	0.001	[0.0001 , 0.01]	0.00999558
$k_{WTasyn2LysUptk}$	0.001 [1]	0.001	[0.0001 , 0.01]	0.00995312

1. Proctor CJ, Tangeman PJ, Ardley HC: **Modelling the role of UCH-L1 on protein aggregation in age-related neurodegeneration**. *Plos One* 2010, **5**:e13175.

2. Martinez-Vicente M, Talloczy Z, Kaushik S, Massey AC, Mazzulli J, Mosharov EV, Hodara R, Fredenburg R, Wu D-C, Follenzi A, Dauer W, Przedborski S, Ischiropoulos H, Lansbury PT, Sulzer D, Cuervo AM: **Dopamine-modified alpha-synuclein blocks chaperone-mediated autophagy**. *J Clin Invest* 2008, **118**:777–788.

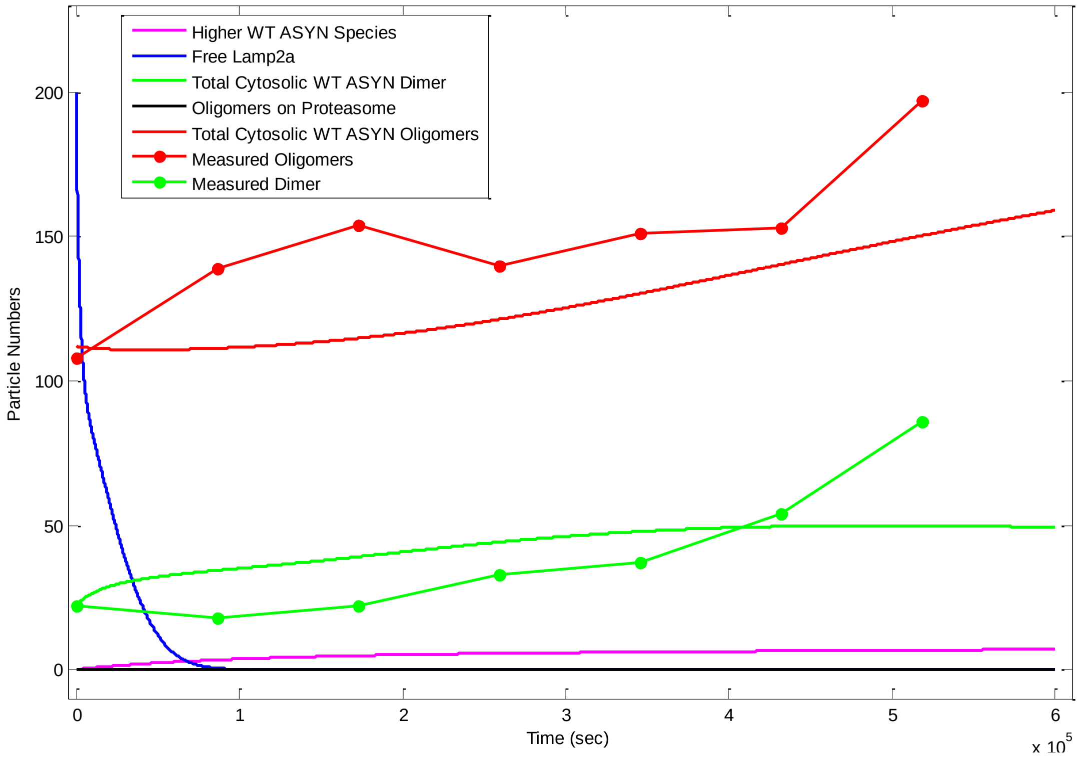

Figure 1: Model simulation results using parameter values estimated by Genetic Algorithm.

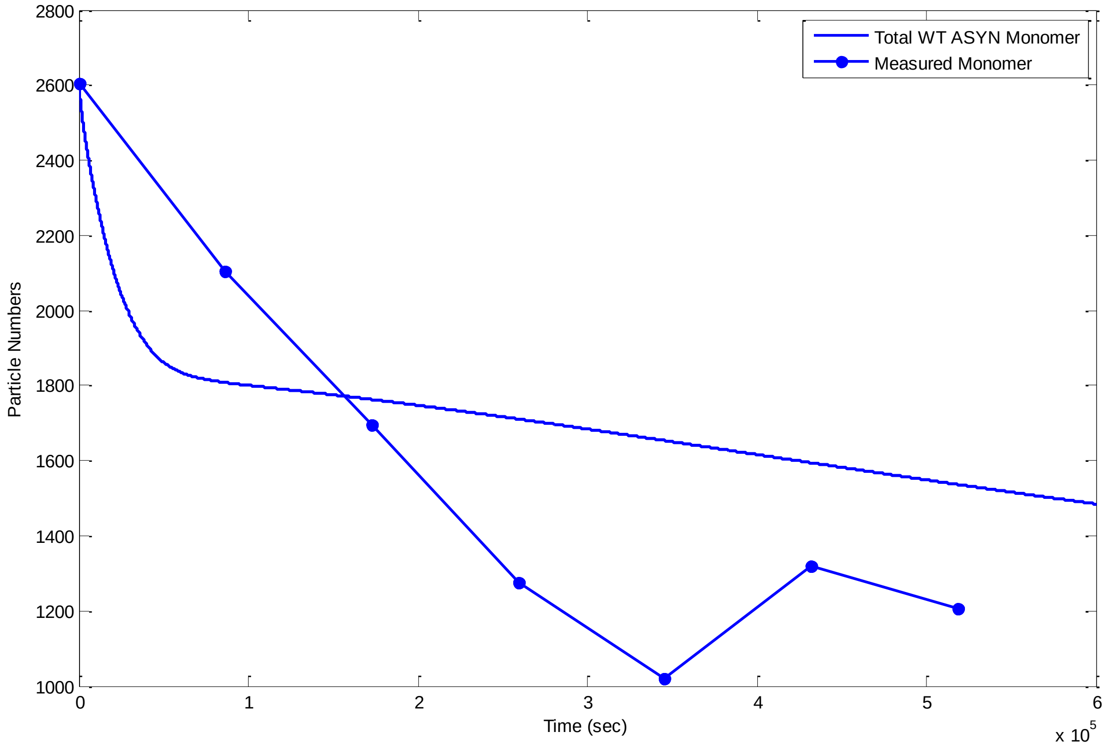

Figure 2: Model simulation results for the monomeric ASYN using parameter values estimated by Genetic Algorithm.

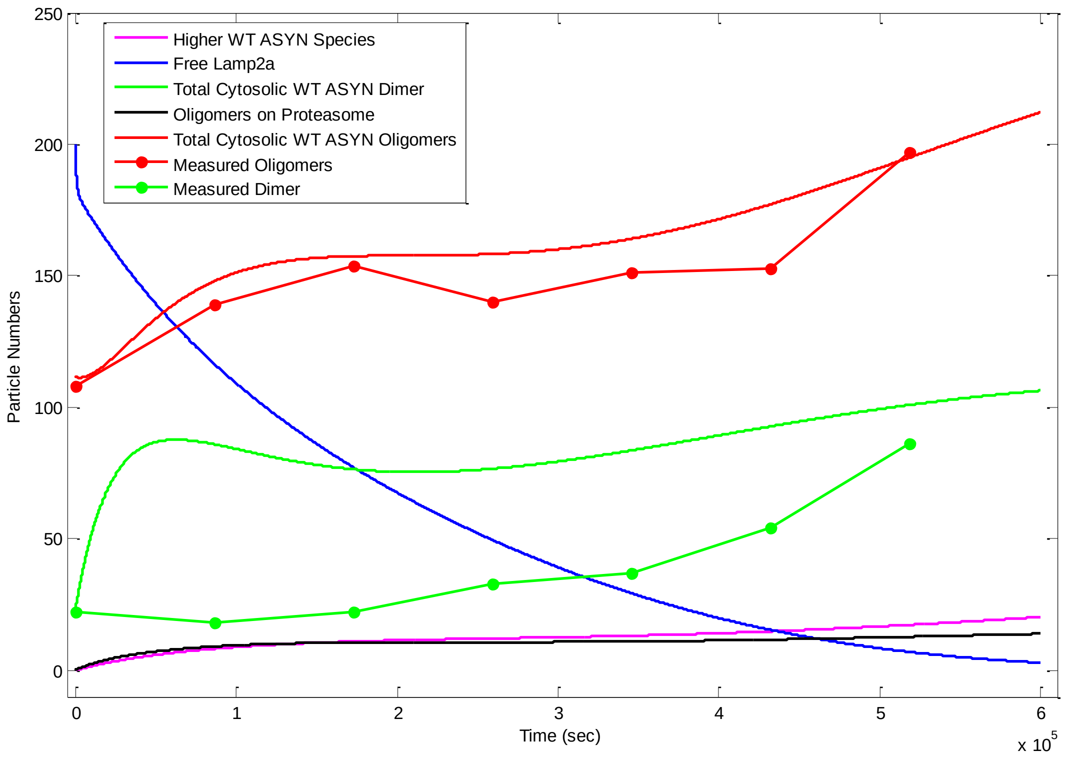

Figure 3: Model simulation results using parameter values estimated by Genetic Algorithm with Stochastic Ranking.

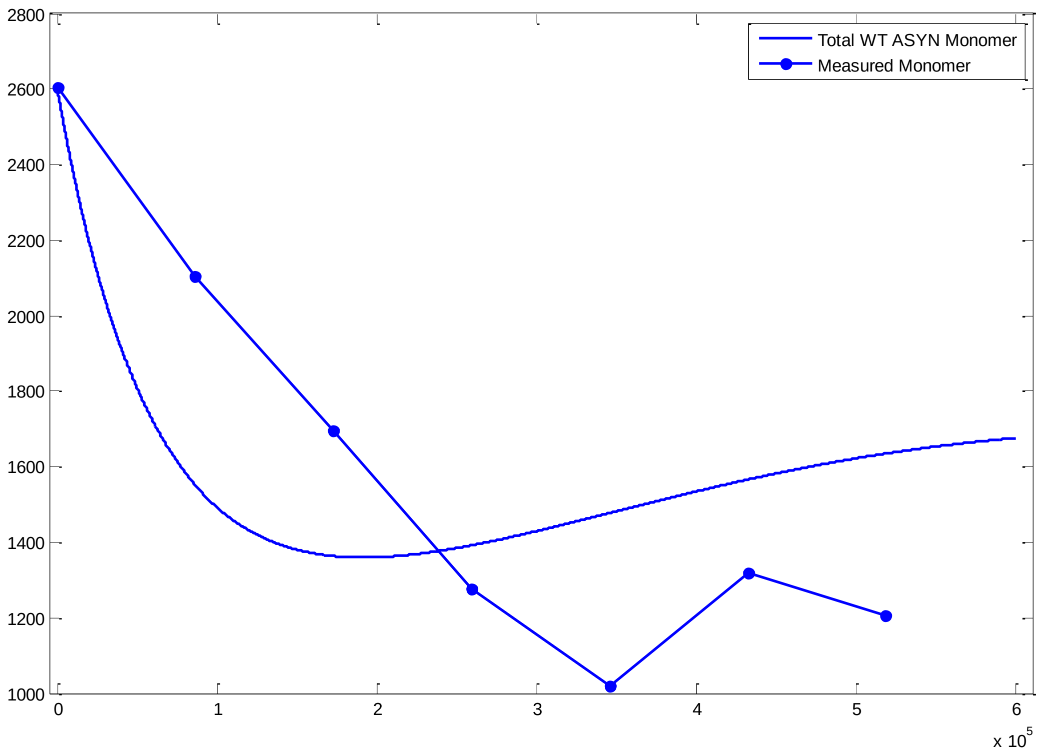

Figure 4: Model simulation results for the monomeric ASYN using parameter values estimated by Genetic Algorithm with Stochastic Ranking.

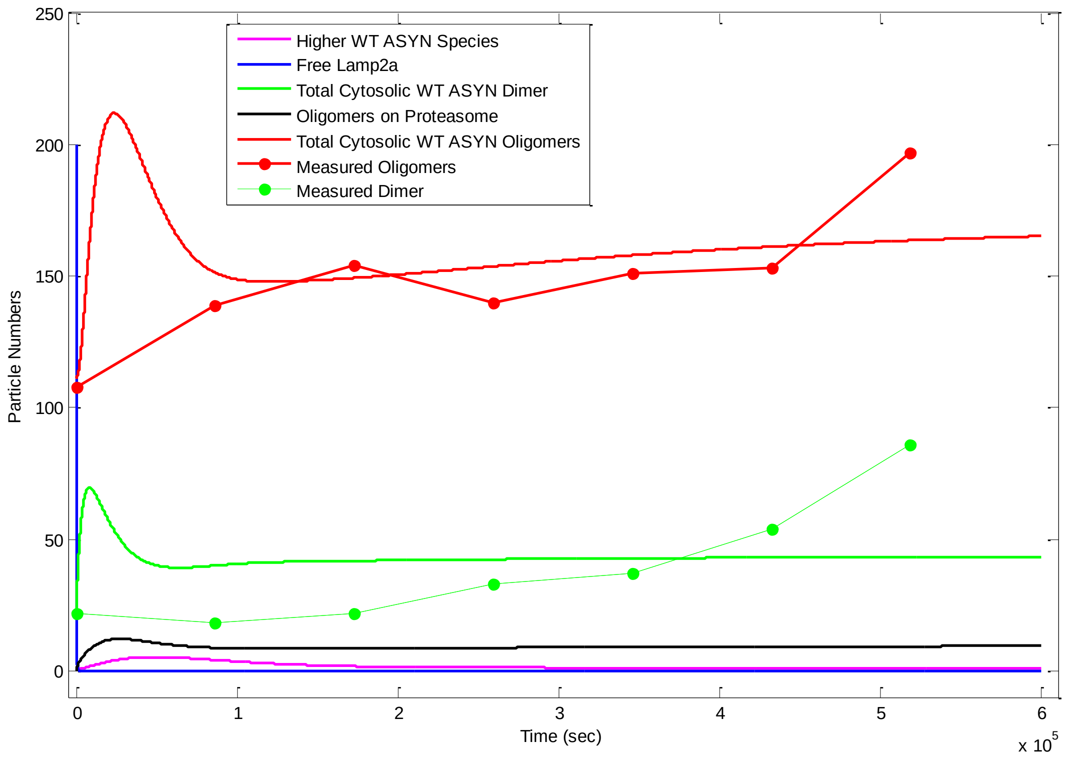

Figure 5: Model simulation results using parameter values estimated by Evolution Strategy algorithm (SRES).

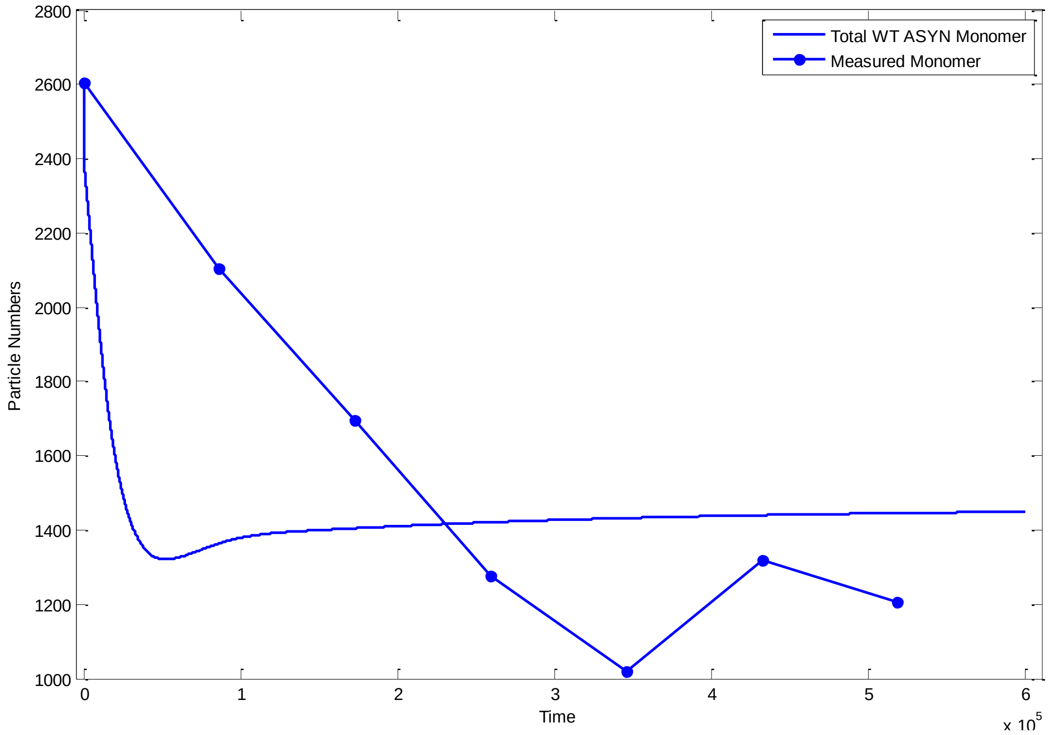

Figure 6: Model simulation results for the monomeric ASYN using parameter values estimated by Evolution Strategy algorithm (SRES).

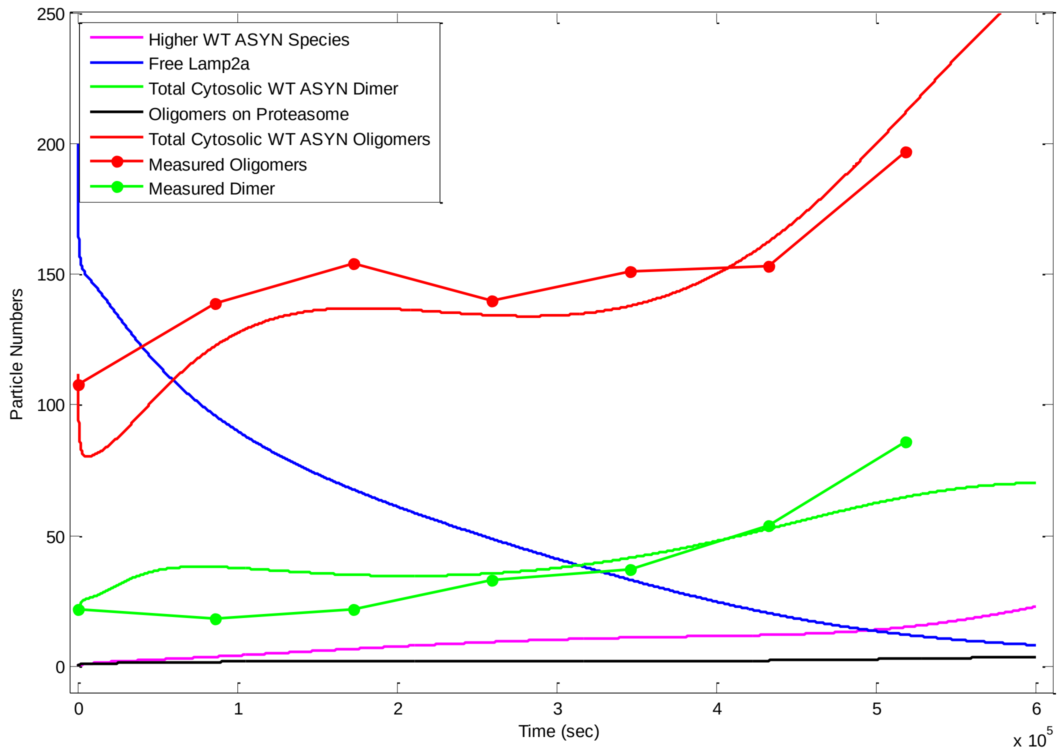

Figure 7: Model simulation results using parameter values estimated by Particle Swarm algorithm.

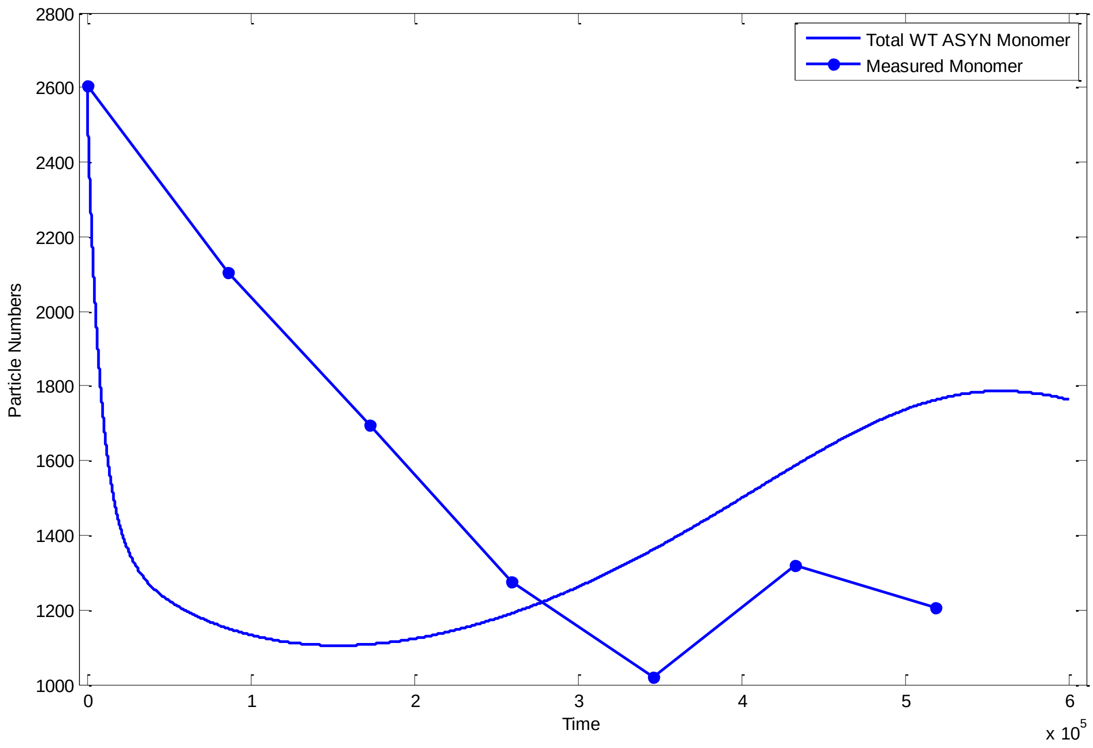

Figure 8: Model simulation results for the monomeric ASYN using parameter values estimated by Particle Swarm algorithm.
