# Supplementary material for: In silico modeling of the effects of alpha-synuclein oligomerization on dopaminergic neuronal homeostasis
Source: BMC Syst Biol. 2014 May 13;8:54. doi: 10.1186/1752-0509-8-54 (PMC4062111; doi:10.1186/1752-0509-8-54)
Supplement: Additional file 6 — Sensitivity analysis results. A table summarizing the results of scaled sensitivity analysis as calculated in COPASI using the available experimental data. [file 1752-0509-8-54-S6.pdf]

Table 1: Sensitivity Analysis Results

	Sensitivity values (scaled sensitivities)		
**Parameter Name**	**Criterion I: Total levels of WT ASYN monomers**	**Criterion II: Total levels of WT ASYN dimers**	**Criterion III: Total sum of WT ASYN oligomers (3-9mers)**
$k_{WTasynSynth}$	0,574409000000	0,767473000000	1,35056000000
$k_{WTasynDopMod}$	0,156937000000	0,588037000000	0,36321600000
$k_{WTasyn2merForm}$	0,141511000000	0,169186000000	0,33177100000
$k_{DopProd}$	0,050302000000	0,156937000000	0,24167100000
$k_{DopModWTasynLampBind}$	0,041930100000	0,110639000000	0,23189400000
$k_{WTOligoBindOnLamp}$	0,002102700000	0,058108900000	0,00021177200
$k_{DopModM/ADegr}$	0,000380270000	0,003127600000	0,00000337801
$k_{WTasyn2merLysDegr}$	0,000267912000	0,000432264000	-0,00000162136
$k_{WTasynLysDegr}$	0,000267309000	0,000415798000	-0,00000309348
$k_{DisRate}$	0,000173683000	0,000383557000	-0,00002257980
$k_{DopMod2merM/ADegr}$	0,000001971630	0,000124558000	-0,00002565350
$k_{WTasyn2LysUptk}$	-0,000000429457	0,000006129670	-0,00018962200
$k_{LampFreeWTasyn}$	-0,000000792020	0,000000388696	-0,00022365700
$k_{WTasynLysUptk}$	-0,000003158690	-0,000000438594	-0,00084612900
$k_{ProtOligDegr}$	-0,000265849000	-0,000014362700	-0,00252914000
$k_{AggrGrowth}$	-0,005391830000	-0,000382850000	-0,01124050000
$k_{OligomerForm}$	-0,006621620000	-0,007667180000	-0,18485500000
$k_{WTasyn1\_2merBindOnLamp}$	-0,156613000000	-0,057112400000	-0,25235900000
$k_{ProteasomeBind}$	-0,161592000000	-0,214919000000	-0,29913200000
$k_{DopDegr}$	-0,168131000000	-0,359317000000	-0,36045800000
$k_{OligAutophagUptake}$	-0,336953000000	-0,980309000000	-0,84454700000
